# Childhood trauma, life-time self-harm, and suicidal behaviour and ideation are associated with polygenic scores for autism

**DOI:** 10.1038/s41380-019-0550-x

**Published:** 2019-10-29

**Authors:** Varun Warrier, Simon Baron-Cohen

**Affiliations:** Department of Psychiatry, Autism Research Centre, University of Cambridge, Cambridge, UK

**Keywords:** Autism spectrum disorders, Genetics

## Abstract

Autistic individuals experience significantly elevated rates of childhood trauma, self-harm and suicidal behaviour and ideation (SSBI). Is this purely the result of negative environmental experiences, or does this interact with genetic predisposition? In this study we investigated if a genetic predisposition for autism is associated with childhood trauma using polygenic scores (PGS) and genetic correlations in the UK Biobank (105,222 < *N* < 105,638), and tested potential mediators and moderators of the association between autism, childhood trauma and SSBI. Autism PGS were significantly associated with childhood trauma (max *R*^2^ = 0.096%, *P* < 2 × 10^−16^), self-harm ideation (max *R*^2^ = 0.108%, *P* < 2 × 10^−16^), and self-harm (max *R*^2^ = 0.13%, *P* < 2 × 10^−16^). Supporting this, we identified significant genetic correlations between autism and childhood trauma (*r*_g_ = 0.36 ± 0.05, *P* = 8.13 × 10^−11^), self-harm ideation (*r*_g_ = 0.49 ± 0.05, *P* = 4.17 × 10^−21^) and self-harm (*r*_g_ = 0.48 ± 0.05, *P* = 4.58 × 10^−21^), and an over-transmission of PGS for the two SSBI phenotypes from parents to autistic probands. Male sex negatively moderated the effect of autism PGS on childhood trauma (*β* = −0.023 ± 0.005, *P* = 6.74 × 10^−5^). Further, childhood trauma positively moderated the effect of autism PGS on self-harm score (*β* = 8.37 × 10^−3^ ± 2.76 × 10^−3^, *P* = 2.42 × 10^−3^) and self-harm ideation (*β* = 7.47 × 10^−3^ ± 2.76 × 10^−3^, *P* = 6.71 × 10^−3^). Finally, depressive symptoms, quality and frequency of social interactions, and educational attainment were significant mediators of the effect of autism PGS on SSBI, with the proportion of effect mediated ranging from 0.23 (95% CI: 0.09–0.32) for depression to 0.008 (95% CI: 0.004–0.01) for educational attainment. Our findings identify that a genetic predisposition for autism is associated with adverse life-time outcomes, which represent complex gene-environment interactions, and prioritizes potential mediators and moderators of this shared biology. It is important to identify sources of trauma for autistic individuals in order to reduce their occurrence and impact.

## Introduction

Autistic individuals have elevated rates of self-harm (with or without suicidal intent) and suicidal behaviour and ideation (SSBI) [1–6]. In addition, there is a positive association between autistic traits (subclinical manifestation of autism features) and SSBI [3, 7]. Suicide is one of the leading causes of mortality in autistic individuals, with the relative risk being higher for autistic women than autistic men [1] (a reversal of the sex ratio in the general population) [8]. There is thus an urgent need to understand and address SSBI in autistic individuals. Despite this, only a handful of studies have investigated variables that contribute to and mediate this [3, 9, 10]. These studies have identified variables such as the stress of camouflaging in autism, depression, lack of social support, and unmet support needs as contributing to SSBI in autistic individuals [3, 9, 10].

A separate line of research has identified that autistic individuals are more prone to childhood trauma (also sometimes referred to as childhood maltreatment, adverse childhood experiences, or childhood abuse and neglect) [11–16]. Autistic traits in the general population are positively correlated with childhood trauma [13, 17]. Childhood trauma and the related sequelae are associated with poor mental and physical health in later life [18–21], and with increased mortality [22–24]. Specifically, childhood trauma is a significant risk factor for SSBI [19, 25–27]. In the general population, childhood trauma accounts for 16–50% [28] of the variance in suicidal ideation, and 64–80% [29] of the variance in suicide attempts, although these studies were conducted in relatively modest sample sizes. In a larger nation-wide registry study of Danish children born in 1966, childhood abuse had the second largest effect on suicide attempt, after a history of psychiatric illness [26]. Thus, childhood trauma is both elevated in autism and, in the general population, is an important risk factor for SSBI. However, it is unclear if elevated childhood trauma interacts with the genetic propensity for autism to increase the risk for SSBI i.e. a diathesis-stress model for SSBI [30].

Autism is highly heritable, with twin and family-based heritability estimates of 60–90% [31–34]. There is also compelling evidence that autism can be modelled as the extreme end of subclinical manifestations of autism, termed autistic traits, which are typically normally distributed in the general population [35–38]. While de novo protein truncating variants in specific genes and CNVs are robustly associated with autism [39–42], 11–49% [43–45] of the variance in autism is attributable to common genetic variants (minor allele frequency > 1%). Polygenic scores (PGS) derived from genome-wide association studies (GWAS) of autism, which represent the underlying genetic predisposition for autism, currently explain a small proportion of the variance in autism (2.5% of the total variance compared to a SNP heritability (*h*^2^_SNP_) of 11%). However, they are a useful index of the genetic predisposition for autism and have been associated with a number of traits in the general population: social and communication difficulties in childhood [46], autistic traits [47], and cognitive aptitude [48]. Importantly, they can be measured in a cohort with genetic data but limited phenotypic data on autism or autistic traits, such as the UK Biobank, and can be used to identify life-term outcomes associated with the genetic predisposition for complex conditions like autism, which may be difficult to investigate in existing autism cohorts. In parallel, recent advances in genetic methods such as genetic correlation [49] and genomic structural equation modelling [50], allow us to understand the shared genetics between various complex traits while accounting for the genetic effects of a third trait. These methods have the added advantage of accounting for greater variance in the genetic propensity for a complex trait compared to currently available PGS.

Both trauma [51, 52] and SSBI [53, 54] are modestly heritable. Specifically, for the childhood trauma phenotypes included in this study, the twin heritability ranged from 0.47 to 0.64 across the different factors included in the childhood trauma questionnaire [55]. Similarly, suicidal ideation has twin heritability ranging from 43% [56] to 57% [57]. A recent GWAS of suicidal attempt in individuals with and without mental health conditions identified a SNP heritability (*h*^2^_SNP_) of 4.6% (95% CI: 2.9–6.3%) [58]. Specifically, the *h*^2^_SNP_ for suicidal attempt for individuals with an autism diagnosis was 9.6% (95% CI: 1.1–18.1%) [58], suggesting a small but significant common genetic component for suicide attempt in autistic individuals. The heritability of both childhood trauma and SSBI represent complex gene-environment effects. For instance, the heritability of childhood trauma may be due to heritable personality traits such as social naivete [59] or risk-taking behaviour [60], which may increase the probability of traumatic experiences in childhood (active gene-environment effect), or where specific personality traits may elicit specific behaviour from family or friends (for example, introverted children are more likely to be bullied, an example of evocative gene-environment effect) [61], or equally where parents have elevated genetic risk for abusive behaviour (passive gene-environment effect). However, while there is a small heritable component to these phenotypes, the importance of the environment cannot be overstated in modifying this genetic predisposition. For instance, an individual with higher genetic predisposition for social naivete or risk-taking behaviour may not experience a traumatic event if they are in a supportive and friendly social environment. In other words, the modest heritability for these phenotypes should not amount to ‘victim blaming’ as different environments may result in very different outcomes.

While previous studies have considerably advanced our understanding of the association between autism and both childhood trauma and SSBI, these studies have almost entirely investigated these associations separately, despite considerable evidence that childhood trauma is a significant risk factor for SSBI. None of these studies have investigated if childhood trauma interacts with autism or autistic traits to increase SSBI. Further, to our knowledge, no study has investigated if the genetic propensity for autism is associated with either childhood trauma or SSBI. As genetic propensity for autism is fixed at birth, they are less likely to be confounded by reverse association. For instance, it is unlikely that autistic traits measured later in life may be influenced by childhood trauma, SSBI, or related sequalae which may confound association analyses between autistic traits and these measures [62]. Recently developed statistical methods also allow us to account for the genetic propensity for other co-occuring conditions such as ADHD, depression, and schizophrenia while investigating the association between the genetic propensity for autism and both childhood trauma and SSBI. This is difficult but not impossible in epidemiological studies as autism is typically diagnosed in childhood and conditions like schizophrenia and depression are typically diagnosed in adults and adolescents. Accounting for the genetic propensity for other, unmeasured conditions such as ADHD and schizophrenia, will allow to better delineate the association between the genetic propensity for autism and childhood trauma and SSBIs.

**Fig. 1:**
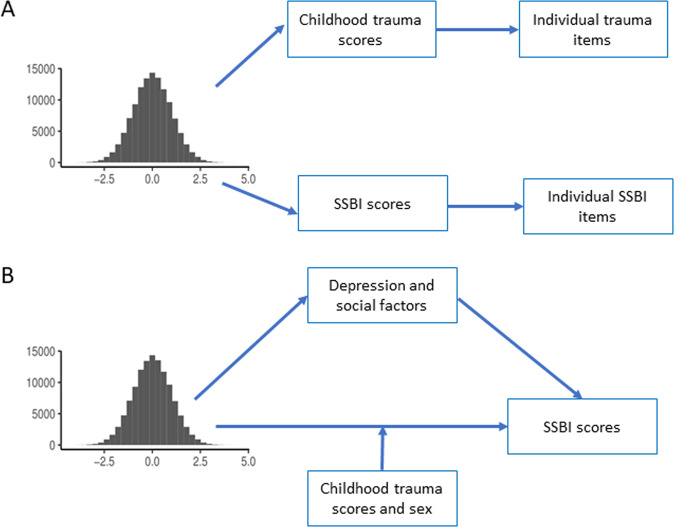
Schematic diagram of the study design. **a** Schematic diagram illustrating the polygenic score association analyses. We generated polygenic scores in the UK Biobank for autism and tested the association between the polygenic scores and childhood trauma scores and SSBI scores. Additionally, to gain deeper insights, we also tested association for individual trauma and SSBI items. As a negative control, we tested for association between Alzheimer’s polygenic scores and both childhood trauma and SSBI scores. We did not test for association between individual items and Alzheimer’s polygenic scores as they were not significantly associated with childhood trauma and SSBI scores. **b** Schematic diagram illustrating the mediation analyses and moderation analyses conducted. Mediation analyses were conducted to investigate if depression and three social factors mediate the association between autism polygenic scores and SSBI scores. Moderation (interaction) analyses were conducted to investigate if sex and childhood trauma influenced the strength of the relationship between autism polygenic scores and SSBI scores

The availability of a large, ageing cohort such as the UK Biobank with rich phenotypic data allows to address these questions. Specifically, in this study, we investigate (Fig. 1): (1) Are childhood trauma and life-time SSBI associated with the genetic propensity for autism? (2) Does the genetic propensity for co-occuring conditions such as ADHD, schizophrenia, and depression affect the association between the genetic propensity for autism and childhood trauma and SSBI? (3) Do sex and childhood trauma moderate the effect of autism PGS on SSBI? (4) Do social, occupational, educational, and neuropsychiatric variables mediate the effects of autism PGS on SSBI?

## Methods

### Participants

Participants were unrelated individuals from the UK Biobank [63] (Year of birth: 1936–1970) of European ancestries [64] as identified using multidimensional scaling and self-report (UK Biobank data field 22006). We restricted our analyses to this group of participants as the GWAS for autism has been conducted in individuals of European ancestries and may not be accurate in individuals of other ancesitries [65]. We excluded participants whose genetic sex did not match their reported sex (sex is used as a covariate in the analyses and we may not be able to covary this correctly in the analysis without this restriction), who were outliers for genetic heterozygosity, and who did not complete the mental health questionnaire [66] (final *N* = 105,638 participants; 44% males). We identified 150 autistic individuals using the UK Biobank data field 20544 (‘Mental Health problems ever diagnosed by a professional’).

### Primary phenotypes

The primary phenotypes used in the study are cumulative scores on measures of childhood trauma and life-time SSBI [66] (Histograms in Supplementary Figs. 1–3):

### Childhood trauma (*N* = 105,638)

Trauma (adult and childhood) was measured using 21 questions, which included five questions for childhood trauma. The five questions were from the childhood trauma screener (CTS), a retrospective measure of trauma designed for adults and adolescents [67]. The CTS has good internal consistency [*α* = 0.757] [67], correlates well with the scales of the longer Childhood Trauma Questionnaire [68], and covers physical, emotional, and sexual abuse, and physical and emotional neglect. Questions were scored from 0 to 4, with options ranging from ‘never true’ to ‘very often true’. We excluded participants who reported ‘prefer not to answer’. For two of the positive items, we inverse scored it to capture trauma. Total scores ranged from 0 to 20, with higher scores representing higher trauma. We used total score as there is evidence to suggest that the total sum of childhood trauma is a better marker of risk for adverse outcomes than individual items [69]. We refer to this phenotype as ‘childhood trauma score’ throughout the results. The items included are:

- Felt loved as a child (inverse scored) (emotional neglect)
- Someone to take me to the doctor as a child (inverse scored) (physical neglect)
- Sexually molested as a child (sexual abuse)
- Physically abused by family as a child (physical abuse)
- Felt hated by family member as a child (emotional abuse)

### SSBI (*N* = 105,222)

In contrast to childhood trauma, the UK Biobank mental health working group did not identify an adequate previously-published instrument to measure SSBI [66]. Self-harm was thus measured using 10 questions in the UK Biobank (UK Biobank data showcase category 146). Three of these questions asked about SSBI in the past year, and we excluded this to focus on life-time self-harm behaviours. We further excluded two questions: ‘Methods of self-harm’, and ‘Action taken following self-harm’, as these cannot be easily included in a scale of SSBI. Finally, we excluded two additional questions: ‘Number of times self-harmed’ and ‘Ever attempted suicide’ as these were completed by only 6,872 participants. Thus, we used four questions of SSBI which were measured on different scales. The first two items had three options: ‘No’, ‘Yes, Once’, ‘Yes, more than once’. The third item had four options: ‘Not at all’, ‘Several days’, ‘More than half the days’, ‘Nearly every day’.

- Ever thought life was not worth living (range: 0–2)
- Ever contemplated self-harm (range: 0–2)
- Recent thoughts of suicide or self-harm (range: 0–3)
- Ever attempted self-harm (binarized: 1 = yes, 0 = no)

Given the range in scores, we constructed two scales, the first being the self-harm ideation scale, which was created by summing up the scores for the first three items. The total score on the self-harm ideation scale ranged from 0 to 7. We refer to this phenotype as ‘self-harm ideation score’. We created a second scale by including all items. For this, we binarized scores for all four items with 1 representing ‘yes’ and 0 representing ‘no’. Thus, the total score on the self-harm scale ranged from 0 to 4. We refer to this phenotype as ‘self-harm score’. For both measures, we excluded participants who chose ‘Prefer not to answer’. Total scores were created only for participants who responded to all the items included in the scores.

### Mediators and moderators of self-harm

We considered the effects of nine measures as mediators of autism PGS and self-harm:

1. Depressive symptoms (39,479 < *N* < 39,551)
2. Anxiety symptoms (28,177 < *N* < 28,231)
3. Friendship dissatisfaction (56,704 < *N* < 56,842)
4. Family relationship dissatisfaction (56,704 < *N* < 56,842)
5. Job dissatisfaction (30,533 < *N* < 30,575)
6. Frequency of friendship/family visits (117,616 < *N* < 117,772
7. Confiding relationship (115,402 < *N* < 115,553)
8. Cognitive aptitude (93,811 < *N* < 93,935)
9. Educational attainment (103,279 < *N* < 103,417)

Further details of how these phenotypes were constructed and methods used in mediation analyses are provided in Supplementary Note Section 1. Previous research has provided support for all these variables influencing SSBI, which is provided in Supplementary Note Section 2. Histograms are provided in Supplementary Fig. 4. Prior to mediation, we investigated if the autism PGS are associated with the mediators, and included only those variables that were associated with autism PGS. We tested each mediator independently rather than in parallel or serially as: (1) We are unable to provide causal relationship between the mediators; (2) It is impossible to provide temporal ordering in this cross-sectional dataset; (3) Several of these variables are moderately correlated with each other (Supplementary Table 1).

We considered two variables as moderators of the effect of PGS on SSBI: sex and childhood trauma score. We draw a distinction between moderators and interaction based on Baron and Kenny [70]. In this framework, a mediator is a variable that represents a mechanism through which the independent variable influences the dependent variable (an intermediary variable). In contrast, a moderator is a variable that affects the strength of the relationship between the independent and the dependent variable (effect modifier), and is equivalent to testing an interaction effect. In this framework, we interpret childhood trauma score and sex as moderators rather than a mediator, as any mediating effect is likely due to downstream effects of trauma such as depression and anxiety in line with the diathesis-stress hypothesis. We note that it is not uncommon to test a variable as both mediator and moderator [71–73].

### Statistical analyses

#### Genotype quality control

We used genotype and imputed SNPs from the UK Biobank [64]. Imputed dosages converted to hard-calls using Plink [74]. Calls with uncertainty greater than 0.1 were treated as missing. We restricted our analyses to SNPs with minor allele frequency >1%, with an imputation *r*^2^ > 0.6, with a per-SNP genotyping rate > 90%, and did not have significant deviations from the Hardy-Weinberg Equilibrium (*P* < 1 × 10^−6^). We excluded individuals who were genetically related (KING-estimated kinship > 0.088, equivalent to third-degree relatives), were not of ‘White British’ ethnicity determined by genetic grouping (UKB Data-field 22006), who had discordant reported and genetic sex, who were outliers for genetic heterozygosity, and who had genotyping rate <90%.

### Polygenic score generation and regression analyses

PGS were constructed using a clumping and thresholding algorithm in PRSice 2 [75]. While there are a few methods that improve the variance explained of the PGS compared to clumping and thresholding [76–79], we decided not to use these as: (1) The increase in variance explained is minimal compared to clumping and thresholding, with one study showing no statistically significant difference in variance explained [80]; (2) The current study investigates covariance rather than variance (i.e. a function of genetic correlation rather than a function of *h*^2^_SNP_), and it is unclear if other methods improve the covariance explained; (3) The large sample size of the testing dataset (UK Biobank) used in the current study makes using methods such as LDPred [76] computationally inefficient and impractical; and 4. We were specifically investigating the shared genetics between autism and childhood trauma and SSBI, making multi-phenotype polygenic scoring methods [78, 79] unsuitable for this study.

PGS are weighted averages of common risk polymorphisms that represent an individual’s inherited propensity for a condition. Weights are assigned for each allele based on the regression *β* value of the GWAS (base dataset), and individuals are scored according to the number of trait-increasing alleles they have (0, 1, or 2). The base dataset was the largest autism GWAS meta-analysis based on 18,381 autistic individuals and 27,969 individuals from the general population [43]. As a negative control, we used a second base dataset: a GWAS meta-analysis of Alzheimer’s Disease (17,008 cases and 37,154 controls) [81]. We choose this dataset as a negative control as there is no significant genetic correlation with the autism GWAS (*r*_g_ = 0.04 ± 0.10; *P* = 0.102), the two GWAS have similar sample sizes and statistical power (Mean chi-square: Alzheimer’s = 1.114, Autism = 1.2), and Alzheimer’s disease is a neurological condition with typical onset late in life. Both GWAS datasets are independent of the participants from the UK Biobank in this study.

We clumped SNPs using an LD-based *r*^2^ of 0.2 and a genomic distance of 250 kb, based on current guidelines [82]. PGS were constructed for seven *P*-value thresholds (*P* = 1, 0.75, 0.5, 0.25, 0.1, 0.01, and 0.001, histograms in Supplementary Fig. 5). These thresholds were chosen to balance the signal-to-noise ratio as autism is highly polygenic [43]. The number of SNPs at each threshold is provided in Supplementary Table 2. In addition, for each item in the three primary phenotypes, we conducted individual PGS-based regression analysis using the *P*-value threshold that explained the maximum variance for the primary phenotype that included the item. We conducted regression analyses using standardized PGS as the independent variable, the first 20 genetic principle components, year of birth, sex, and genotyping batch as covariates in the model, all of which were standardized. Linear regression analyses were conducted for all analyses except for the individual items in the SSBI measures as these were binarized, and thus suitable for a logistic regression. For the variables that were significantly associated with autism PGS, we also investigated the average scores of the variables in the top and the bottom centiles of the PGS uncorrected for any covariates.

The UK Biobank has a healthy volunteer bias and participants were born before 1970. As such only 223 out of 50,099 individuals in the UK Biobank reported a diagnosis of autism, when asked as a part of the mental health questionnaire. This (0.4%) is lower than the reported prevalence of autism in the UK and the US (1–2%). The lower prevalence in this cohort may be attributed to both a healthy volunteer bias, and the fact that this is an older cohort, resulting in an underdiagnosis of autism, though empirical evidence suggests that the estimated prevalence of autism (diagnosed and undiagnosed combined), does not vary with age [83]. Given the small number of individuals with an autism diagnosis in the UK Biobank, power calculations indicated that we had only 50% statistical power, and thus were underpowered to investigate if PGS for autism are associated with case-control status in the UK Biobank. We were, thus, unable to test within the UK Biobank if autistic individuals have higher autism PGS. However, studies have tested the association of PGS from the latest iPSYCH-PGC autism GWAS [43], which we use in the current study, and identified a variance explained of 2.45% [43]. In the typical population, autism PGS from the same GWAS, explained 0.13% of variance in social and communication difficulties in children at age 8 [46].

### Polygenic transmission disequilibrium test

We conducted polygenic transmission disequilibrium test (pTDT) [84] in *N* = 2,234 families from the simons simplex collection (SSC) [85], of primarily European Ancestry (identified by multidimensional scaling) to investigate if PGS for the three primary phenotypes are over-transmitted from parents to autistic probands compared to sibling controls. pTDT is a modified *t* test which compares the mean PGS in autistic individuals compared to the mean mid-parent PGS. pTDT is a within-family statistical test, and is less confounded by factors such as population stratification and assortative mating. Details of QC in the SSC are provided in the Supplementary Note section 3. We constructed PGS at *P* = 1 as these phenotypes are highly polygenic. PGS were constructed using PRSice as outlined earlier.

### GWAS, genetic correlation analyses, and genomic SEM

To provide further support to the results of the PGS analyses we conducted GWAS of the three primary phenotypes, details of which are provided in the Supplementary Note section 4. We conducted genetic correlation between autism and the three primary phenotypes using LDSC [49, 86]. LD scores were generated using a north-west European population. To better understand the shared genetics between autism and the three primary phenotypes after accounting for the common genetic effects of various co-occuring conditions, we conducted genomic structural equation modelling (SEM) analyses [50]. We used genome-wide summary statistics for:

1. ADHD [87]: *N* = 20,183 cases and 35,191 controls
2. Major Depressive Disorder [88]: *N* = 59,851 cases and 113,154 controls (excluding 23andMe)
3. Schizophrenia [89]: *N* = 40,675 cases and 64,643 controls
4. Cognitive aptitude [90]: *N* = 257,828
5. Educational attainment [90]: *N* = 766,345 (sample size after excluding data from 23andMe)

These GWAS summary statistics were chosen keeping in mind their modest/high genetic correlation with autism, and the mean sample size.

### Mediation and moderation analyses

We modelled interaction between sex and PGS, and childhood trauma scores and PGS. We further conducted a series of mediation analyses to identify potential variables that mediate the association between autism PGS and SSBI. All variables were standardized for both the moderation and the mediation analyses. For the moderation analysis with SSBI as the dependent variable, and for all mediation analyses, we restricted our investigations to a PGS P-value threshold of 0.75 as this explained the maximum variance in SSBI. For the moderation analysis with childhood trauma as the dependent variable, we used a P-value threshold of 1 as this explained the maximum variance in childhood trauma.

### Multiple testing correction

For each analysis conducted, we corrected for the multiple tests conducted using Bonferroni correction.

1. Regression analyses for the primary outcome variables: *P* = 2 × 10^−3^ (seven *P*-value thresholds for three primary phenotypes).
2. Item-level analysis: *P* = 5.5 × 10^−3^ (nine individual items investigated).
3. pTDT: *P* = 0.0167 (3 tests conducted).
4. Genetic correlation: *P* = 0.0167 (three tests conducted).
5. Genomic SEM: *P* = 0.0034 (5 × 3 tests conducted).
6. Interaction analyses: *P* = 0.01 (three sex*PGS interaction tests conducted, and 2 childhood trauma score*PGS interaction tests).
7. Mediation analyses: Only five variables of the nine tested were associated with autism PGS. Thus, we identify significant results at a Bonferroni corrected *α* of *P* = 5 × 10^−3^.

## Results

### Autistic individuals in the UK Biobank have elevated rates of all three phenotypes

We first investigated if autistic individuals (*N* = 150, ‘Methods’) identified in the UK Biobank have elevated rates of childhood trauma and the two SSBI phenotype, after covarying for the effects of age and sex. We identified substantially elevated mean scores for childhood trauma (*β* = 0.98 ± 0.08, *P* < 2 × 10^−16^), self-harm ideation (*β* = 1.18 ± 0.08, *P* < 2 × 10^−16^), and self-harm scores (*β* = 1.05 ± 0.08, *P* < 2 × 10^−16^), confirming, in the UK Biobank, previous findings.

### Autism PGS are associated with childhood trauma

**Fig. 2:**
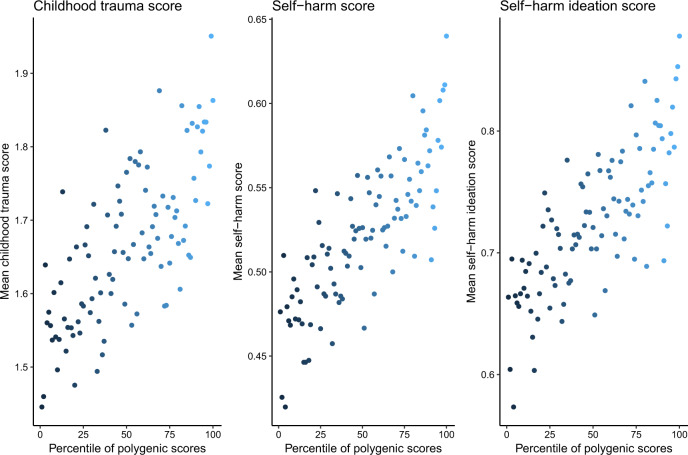
Scores on childhood trauma and SSBI based on centiles of PGS. **a**. This figure provides the scores of three primary phenotypes against the percentile of PGS after the cohort was divided into 100 groups based on PGS. Each dot in the plot represents an average phenotypic score for that group. Colours indicate the gradient of percentile, with lighter blue representing the highest percentile and darker blue representing the lowest percentile

We investigated if autism PGS are associated with childhood trauma. PGS at all seven *P*-value thresholds were significantly associated with childhood trauma score (Supplementary Fig. 1), with the highest variance at *P* = 1 (300,133 SNPs, *R*^2^ = 0.096%, *P* < 2 × 10^−16^) (Table 1). In contrast, across the seven *P*-value thresholds, PGS for Alzheimer’s were not significantly associated with childhood trauma scores (Supplementary Table 3). Dividing the cohort into centiles based on autism PGS (*P* = 1), the top 1% had, on average, a 28% increase in childhood trauma scores compared to the bottom 1% (Fig. 2).

**Table 1:** Effect of PGS for autism across the three primary phenotypes

*β*	SE	*Z*	*P*	*P*-threshold	*R*^2^	Phenotype
3.11E−02	2.88E−03	10.784	<2.2E−16	1	0.0958	Childhood trauma
3.11E−02	2.88E−03	10.769	<2.2E−16	0.75	0.0955	Childhood trauma
3.07E−02	2.88E−03	1.06E+01	<2.2E−16	0.5	0.0932	Childhood trauma
3.06E−02	2.88E−03	1.06E+01	<2.2E−16	0.25	0.0926	Childhood trauma
2.82E−02	2.88E−03	9.79E+00	<2.2E−16	0.1	0.0787	Childhood trauma
2.23E−02	2.88E−03	7.72E+00	1.22E−14	0.01	0.0486	Childhood trauma
1.24E−02	2.88E−03	4.287	1.82E−05	0.001	0.0144	Childhood trauma
3.28E−02	2.86E−03	1.14E+01	<2.2E−16	1	0.106	Self-harm ideation
3.31E−02	2.86E−03	1.16E+01	<2.2E−16	0.75	0.108	Self-harm ideation
3.28E−02	2.86E−03	1.15E+01	<2.2E−16	0.5	0.107	Self-harm ideation
3.12E−02	2.86E−03	1.09E+01	<2.2E−16	0.25	0.096	Self-harm ideation
2.98E−02	2.86E−03	1.04E+01	<2.2E−16	0.1	0.088	Self-harm ideation
2.58E−02	2.86E−03	9.01E+00	<2.2E−16	0.01	0.066	Self-harm ideation
1.53E−02	2.86E−03	5.32E+00	1.00E−07	0.001	0.023	Self-harm ideation
3.61E−02	2.86E−03	1.26E+01	<2E−16	1	0.129	Self-harm score
3.63E−02	2.86E−03	1.27E+01	<2E−16	0.75	0.13	Self-harm score
3.61E−02	2.86E−03	1.26E+01	<2E−16	0.5	0.129	Self-harm score
3.48E−02	2.86E−03	1.21E+01	<2E−16	0.25	0.12	Self-harm score
3.31E−02	2.86E−03	1.16E+01	<2E−16	0.1	0.108	Self-harm score
2.77E−02	2.86E−03	9.66E+00	<2E−16	0.01	0.075	Self-harm score
1.74E−02	2.86E−03	6.08E+00	1.22E−09	0.001	0.029	Self-harm score

This table provides the result of the polygenic score analyses for the three primary phenotypes at seven different *P*-value thresholds. For each analysis, we report the regression coefficient (*β*) and the accompanying standard errors (SE), *Z*-score (*Z*) and *P*-value of the *Z*-score (*P*). Variance explained (*R*^2^*)* is provided in percentages

To better understand which individual items contribute to the association between autism PGS and childhood trauma, we investigated the association between autism PGS (*P* = 1) and each of the five individual trauma items (Supplementary Fig. 2). For four of the five measures (inverse-scored ‘felt loved as a child’, ‘felt hated as a child’, ‘physically abused’, and ‘sexually molested’), PGS were significantly and positively associated with the traumatic event (Table 2), with the highest variance explained for the inverse-scored item ‘felt loved as child’ (*R*^2^ = 0.092%, *P* < 2 × 10^−16^) and the lowest for ‘sexually molested’ (*R*^2^ = 0.01%, *P* = 9.8 × 10^−5^). Compared to the bottom 1%, the top 1% reported a 77%, 24%, 34%, and 45% increase in scores for the items ‘felt hated as a child’, ‘felt loved as a child’ (inverse scored), ‘physically abused’, and ‘sexually molested’, respectively (Supplementary Fig. 6**)**.

**Table 2:** Effect of PGS on individual phenotypic items

Item	*β*	SE	*Z*	*P*	*P*-threshold	*R*^2^ (%)	Category
Felt loved as child	3.07E−02	2.89E−03	10.60	<2.2E−16	1	0.092	Childhood trauma
Sexually molested as a child	1.12E−02	2.88E−03	3.89	9.85E-05	1	0.01	Childhood trauma
Physically abused as a child	2.53E−02	2.88E−03	8.79	<2.2E−16	1	0.063	Childhood trauma
Felt hated as a child	2.68E−02	2.87E−03	9.33	<2.2E−16	1	0.07	Childhood trauma
Taken to the doctor as a child	1.78E−03	2.88E−03	0.61	0.53	1	0.0004	Childhood trauma
Ever thought life not worth living	3.27E−02	2.86E−03	11.43	<2.2E−16	0.75	0.168	SSBI
Ever contemplated self-harm	3.10E−02	2.86E−03	10.85	<2.2E−16	0.75	0.154	SSBI
Ever attempted self-harm	2.63E−02	2.88E−03	9.12	<2.2E−16	0.75	0.107	SSBI
Recent thoughts of suicide and self-harm	7.87E−03	2.90E−03	2.71	0.00655	0.75	0.01	SSBI

This table provides the result of the polygenic score analyses for the individual items at specific P-value thresholds. For each analysis we report the regression coefficient (*β*) and the accompanying standard errors (SE), *Z*-score (Z) and *P*-value of the *Z*-score (*P*-value). Variance explained (*R*^2^) is provided in percentages. Please note, the variance explained for SSBI items is measured using Nagelkerke’s pseudo *R*^2^ given that these were binary phenotypes

### Autism PGS are associated with SSBI

Autism PGS were also significantly and positively associated with both self-harm ideation and self-harm scores (Table 1 and Supplementary Fig. 1). The variance explained for self-harm ideation was highest at *P* = 0.75 (261,065 SNPs, *R*^2^ = 0.108%, *P* < 2 × 10^−16^). Similarly, for total self-harm scores, the variance explained was highest at *P* = 0.75 (261,065 SNPs, *R*^2^ = 0.13%, *P* < 2 × 10^−16^). In contrast, Alzheimer’s PGS were not associated with either self-harm ideation or self-harm scores at any of the 7 thresholds tested (Supplementary Table 3). Individuals in the top 1% of the autism PGS reported a 34% increase in self-harm scores and a 32% increase in self-harm ideation compared to the bottom 1% **(**Supplementary Fig. 7**)**.

At an item level (Supplementary Fig. 3), autism PGS (*P* = 0.75) were significantly associated with three of the four items (‘thought life not worth living’, ‘contemplated self-harm’, and ‘attempted self-harm’) (Table 2), with the highest variance explained for ‘thought life not worth living’ (Nagelkerke’s pseudo *R*^2^ = 0.17%, *P* < 2 × 10^−16^). Autism PGS were only nominally associated with the item ‘recent thoughts of suicide or self-harm’ (Nagelkerke’s pseudo *R*^2^ = 0.01%, *P* = 0.006). Compared to the bottom 1%, the top 1% reported a 41%, 95%, and 30% increase in scores on the items ‘contemplated self-harm’, ‘attempted self-harm’, and ‘thought life was not worth living’, respectively (Supplementary Fig. 7).

We repeated the associations between autism PGS and the three primary phenotypes and items after excluding autistic individuals. The results were similar and remained statistically significant (Supplementary Table 4).

### Genetic correlation confirms shared heritability between autism, childhood trauma and SSBI

**Fig. 3:**
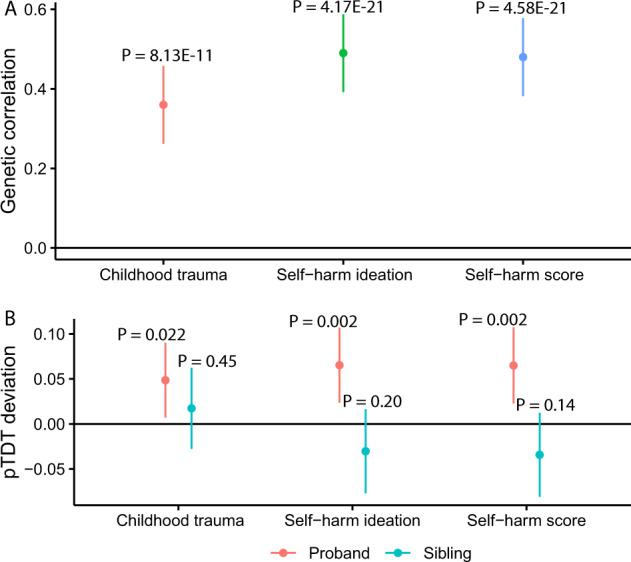
Genetic correlation and pTDT analysis of the three primary phenotypes and autism. **a** This figure provides the results of the genetic correlation analyses between the GWAS for autism and the GWAS for the three primary phenotypes. The dot provides the estimate (genetic correlation), and the lines represent the 95% confidence intervals. The *P*-values are indicated on top of each line. **b** This figure provides the results of the pTDT analyses. The *y*-axis provides the mean pTDT deviation in standard deviations of the mid-parent polygenic scores. Red dot and lines represent the estimate and the 95% CI respectively for the proband (autistic individuals), and the blue dot and lines represent the estimate and the 95% CI respectively for the non-autistic sibling control

To validate the results identified from the PGS analysis, we conducted GWAS of the three primary phenotypes (‘Methods’), and investigated genetic correlations. Both the SSBI phenotypes and childhood trauma had a modest and statistically significant SNP heritabilities (0.071 < *h*^2^_SNP_ < 0.083). Manhattan and QQ plots are provided in Supplementary Figs. 8–10. We identified significant genetic correlations between autism and self-harm ideation (*r*_g_ = 0.49 ± 0.05, *P* = 4.17 ×10^−21^), and self-harm scores (*r*_g_ = 0.48 ± 0.05, *P* = 4.58 × 10^−21^) (Fig. 3a). The genetic correlation between autism and childhood trauma was lower but still statistically significant (*r*_g_ = 0.36 ± 0.05, *P* = 8.13 × 10^−11^) (Fig. 3a). Comparing these with previously reported genetic correlations with autism, we note that the absolute magnitude of genetic correlation between autism and the two SSBI phenotypes is among the highest observed, comparable to that between autism and depression (*r*_g_ = 0.41 ± 0.04, *P* = 1.40 × 10^−25^), and higher than the genetic correlation between autism and schizophrenia (*r*_g_ = 0.21 ± 0.04, *P* = 1.03 × 10^−5^), educational attainment (*r*_g_ = 0.19 ± 0.03, *P* = 2.56 × 10^−9^), and ADHD (*r*_g_ = 0.36 ± 0.05, *P* = 1.24 × 10^−12^) [43].

### PGS for SSBI phenotypes are over-transmitted to autistic probands

While these analyses point to a genetic predisposition to autism and SSBI and childhood trauma, the results are not immune to confounding due to ascertainment issues or heterogeneity. We thus conducted family-based pTDT in 2,234 autism families to investigate if PGS for childhood trauma, and the two SSBI phenotypes are over-transmitted from parents to autistic probands compared to their non-autistic siblings (PGS *P*-value threshold = 1). We identified a significant over-transmission of PGS for self-harm ideation (*P* = 2.2 × 10^−3^) and self-harm scores (*P* = 2.6 × 10^−3^) from parents to their autistic children. For childhood trauma, there was a nominal over-transmission though this was not statistically significant after correcting for the three tests (*P* = 0.022). In contrast, we did not identify a significant over-transmission of PGS for childhood trauma (*P* = 0.45), self-harm ideation (*P* = 0.20), and self-harm score PGS (*P* = 0.14) from parents to non-autistic sibling controls (*N* = 1,829 unrelated sibling controls) (Fig. 3b and Supplementary Table 5).

### Genomic SEM delineates provides further insights into the shared genetics autism and the three primary phenotypes

Given the modest to high shared genetics between autism and multiple other co-occuring conditions and measures of intelligence, we next conducted genomic structural equation modelling (Supplementary Fig. 11) to investigate the genetic correlations between autism and the three primary phenotypes after accounting for the genetic effects of other variables (‘Methods’). For childhood trauma, the genetic correlation with autism was substantially attenuated after accounting for the genetic effects of ADHD (*r*_g_ = 0.14 ± 0.05, *P* = 3.64 × 10^−3^) and depression (*r*_g_ = 0.09 ± 0.06, *P* = 0.10). The genetic correlation attenuated modestly after accounting for the genetic effects of schizophrenia (*r*_g_ = 0.26 ± 0.05, *P* = 2.45 × 10^−6^), and there was minimal attenuation after accounting for the genetic effects of either cognitive aptitude (*r*_g_ = 0.36 ± 0.06, *P* = 1.65 × 10^−10^) or educational attainment (*r*_g_ = 0.37 ± 0.06, *P* = 4.59 × 10^−10^).

For the two SSBI phenotypes, accounting for the genetic effects of depression substantially attenuated the genetic correlation with autism (self-harm ideation: *r*_g_ = 0.15 ± 0.05, *P* = 7.26 × 10^−3^; self-harm score: *r*_g_ = 0.14 ± 0.05, *P* = 0.01). Accounting for the genetic effects of none of the other phenotypes substantially attenuated the genetic correlation between autism and the two SSBI variables (Supplementary Table 6).

### Social variables and depression mediate the effect of PGS on SSBI

We investigated if nine different variables (Supplementary Fig. 4) mediate the relationship between PGS for autism and SSBI. Autism PGS were significantly associated with friendship dissatisfaction, family relationship dissatisfaction, depressive symptoms, frequency of social interactions, cognitive aptitude and educational attainment (Supplementary Table 7, *α* = 0.007). In addition, with the exception of cognitive aptitude, all mediating variables were significantly associated with both the SSBI phenotypes **(**Supplementary Table 8**)**, and thus were taken forward for mediation analyses. All five variables significantly mediated the relationship between autism PGS and the two SSBI phenotypes (Supplementary Table 9). The proportion of mediated effect was highest for depressive symptoms (23%, average causal mediated effect or ACME = 8.9 × 10^−3^ ± 2.57 × 10^−3^) and lowest for educational attainment (0.8%, ACME = 3.05 × 10^−4^ ± 7.55 × 10^−5^).

### Sex and childhood trauma moderate the effect of PGS on SSBI

vFinally, we investigated if sex moderates the effect of autism PGS on childhood trauma scores and SSBI. Across all three measures, the main effects of sex and PGS were significant (Supplementary Table 10). Female sex was significantly and positively associated with higher childhood trauma and SSBI. Sex significantly interacted with PGS to predict childhood trauma score (*β*_Males_ = −0.023 ± 0.005, *P* = 6.74 × 10^−5^), but not self-harm score (*β*_Males_ = −0.014 ± 0.006, *P* = 0.013) or self-harm ideation (*β*_Males_ = −0.012 ± 0.006, *P* = 0.026). To test the diathesis-stress model, we investigated if childhood trauma score significantly interacted with autism PGS. Childhood trauma scores significantly moderated the effects of autism PGS on both self-harm score (*β* = 8.37 × 10^−3^ ± 2.76 × 10^−3^, *P* = 2.42 × 10^−3^) and self-harm ideation (*β* = 7.47 × 10^−3^ ± 2.76 × 10^−3^, *P* = 6.71 × 10^−3^) (Supplementary Table 10).

## Discussion

While autism is often and should be diagnosed in childhood, increasing research has focussed on the long-term mental and physical health of autistic individuals throughout adulthood. Specifically, a growing body of research has identified that autistic adults are at higher risk for SSBI [1–6] and life-long vulnerability [91]. These studies are all limited in that they have tested relatively small cohorts, and have typically relied on clinical groups. We extend these results by modelling the underlying genetic propensity for autism using PGS in more than 100,000 individuals from the UK Biobank.

We find that PGS for autism are significantly associated with both childhood trauma and SSBI and several individual items contributing to these measures, which increase continuously along a gradient of increasing PGS for autism. In contrast, we do not observe an association between PGS for Alzheimer’s and the three primary phenotypes, suggesting that the observed results are not just a function of the large sample size in the UK Biobank. Dividing the cohort into centiles of PGS demonstrates a sharp increase in SSBI and childhood trauma between the top and bottom 1%. These results are supported by substantial genetic correlation between autism and the three primary phenotypes. Notably, the genetic correlation between autism and the two SSBI phenotypes is higher than the genetic correlation between autism and ADHD, major depression, and schizophrenia [43].

These results are not immune to confounding due to multiple factors. For instance, fine-scale population stratification cannot be completely accounted for using current methods [92]. Further, there may be systematic differences between autistic individuals and non-autistic individuals that may be unrelated to an autism diagnosis, though this is minimized by the iPSYCH study design which should not have a recruitment bias [93]. However, using a family-based association technique we confirm the robustness of the results—we identify a significant over-transmission of PGS for the two SSBI phenotypes from parents to autistic children but not to their non-autistic siblings. The results for childhood trauma were nominally significant but did not remain significant after correcting for multiple testing, possibly due to the small sample size used to conduct the pTDT and the smaller genetic correlation with autism compared to the two SSBI phenotypes. A potential confounding factor that we could not test in this study is age of diagnosis of autism, since it might be the case that late diagnosis might increase the risk of SSBI, if those diagnosed late had a longer period of their life with no support.

While trauma is thought to be a largely environmental factor, we propose three potential mechanisms by which PGS for autism may be associated with childhood abuse. First, elevated PGS for autism may lead to difficulties in social interaction, communication, and socially inappropriate behaviour [47, 94], which may, in turn, evoke abusive or neglectful behaviours from parents, caregivers, and peers, leading to greater childhood trauma. Second, social naivete among autistic children may contribute to higher exposure to potentially dangerous situations, which may lead to greater incidence of trauma. Finally, evaluating an event as being traumatic depends on an individual’s assessment of the event. This may be particularly pertinent for this study, as childhood trauma was measured retrospectively using a self-report measure and retrospective and prospectively measured trauma are only partly correlated [95]. Some autistic individuals may be sensitive to perceiving an event as traumatic. This does not in any way question the validity of the trauma experienced by autistic individuals, as trauma is not just an event but also how we perceive an event. Finally, the risk of SSBI in autistic teenagers and adults may be because they have experienced exclusion by society, bullying by their peers, ridicule by their teachers, late diagnosis and therefore an absence of early support for autism, and a lack of life-long support, given that autism is a life-long condition. Each of these possible mediating factors must be formally tested.

It is vital to interpret these results correctly. These phenotypes are results of gene-environment interactions. Modifying the environment may alter the outcome (self-harm or childhood trauma). For instance, providing a supportive and inclusive environment to autistic individuals early on may reduce both childhood trauma and SSBI. In other words, the association between genetic predisposition to autism and the three primary phenotypes should not amount to victim blaming as modifying the environment can modify the outcomes. We have included an FAQ as a Supplementary Text to provide further clarity to our findings.

Given the substantial comorbidity and shared genetics between autism and conditions like ADHD, depression, and schizophrenia, and the modest shared genetics between measures of intelligence, we used genomic structural equation modelling to better understand how pleiotropy affects the shared genetics between autism and the three primary phenotypes. Across all three phenotypes, accounting for the genetic effects of depression rendered the genetic correlation with autism statistically non-significant. Further, for childhood trauma, accounting for ADHD substantially attenuated the genetic correlation with autism, and the genetic correlation was no longer statistically significant. These results suggest that the shared genetic component between autism and depression underlies the association of genetic predisposition to autism and the three primary phenotypes. We caution that there may be a recall bias that may confound these analyses as depressed individuals may be more likely to recall traumatic events, and these results must be interpreted accordingly [96].

While it is well established that childhood trauma contributes to SSBI in later life in the typical population, it is unclear how this interacts with autism or autistic traits. Our results suggest that there is a significant interaction between PGS for autism and childhood trauma to contribute to increased SSBI, providing evidence for a gene-by-environment interaction. We note that the P-values are modest for a cohort the size of the UK Biobank, and these results must be replicated in a large, independent cohort and using PGS derived from an independent GWAS of autism when available. If replicated, for individuals with high PGS for autism, this represents a ‘double hit’. Not only are high PGS for autism associated with higher SSBI in adulthood, these scores are also associated with higher childhood trauma which also increases the risk for SSBI.

Finally, we identify that depressive symptoms, quality of social relationships (friendship satisfaction and family relationship satisfaction), frequency of social interactions, cognitive aptitude and educational attainment significantly mediate a small proportion of the association between autism PGS and SSBI. While these provide a model for further investigation, we caution careful interpretation of these results. Mediators must typically have temporal precedence over the dependent variable, which we were unable to clearly establish in this study and needs to be investigated using longitudinal models.

While directional correlation is of interest to better understand causality, this study did not test this as the current GWAS of autism is underpowered to develop a genetic instrumental variable for Mendelian Randomization methods, given the number of statistically significant loci identified for autism, childhood trauma, and SSBI. As such, we caution against interpreting these results using a causal framework. The current research only strengthens the epidemiologically identified correlation between autism and both SSBI and childhood trauma, and does not imply causality.

This study has a few limitations, which we have tried to partially address using multiple methods. First, UK Biobank participants are likely to be healthier, better educated and more affluent than the general population [97], suggesting that the rates of self-harm behaviour and childhood trauma may be lower than that in the general population. It is, however, encouraging to observe statistically significant results for the two SSBI PGS using pTDT, as the within-family analyses accounts for some potential confounds. Second, while the GWAS used to construct PGS for autism is the largest to date, it still captures only 2.5% of the total variance compared to a SNP heritability of 11% [43]. In turn, the percentage of variance explained by the regressions, mediation and moderation analyses are also small. However, to remediate this, we additionally conducted statistical analyses using summary GWAS data, which capture a greater proportion of the variance. Third, this study focusses only on common variants even though rare variants and CNVs contribute to a fraction of the variance in autism. Fourth, childhood trauma has been measured retrospectively and this could introduce bias in the measurement of childhood trauma. Recent evidence points to only modest agreement between prospective and retrospective measures of trauma [95], suggesting that the results may not be applicable to prospective measures of trauma.

## Supplementary information

Supplementary FAQ

Supplementary Information (Figures, Tables, and Text)

## Data Availability

All codes used in this analysis are available here: https://github.com/autism-research-centre/Autism_vulnerability_UKB. Data are available from the UK Biobank to approved researchers.
