# Supplementary material for: Childhood trauma, life-time self-harm, and suicidal behaviour and ideation are associated with polygenic scores for autism
Source: Mol Psychiatry. 2019 Oct 29;26(5):1670–84. doi: 10.1038/s41380-019-0550-x (PMC8159746; doi:10.1038/s41380-019-0550-x)
Supplement: Supplementary file 1 — Supplementary FAQ [file 41380_2019_550_MOESM1_ESM.docx]

**FAQs**

**Childhood trauma, life-time self-harm and suicidal behaviour and ideation are associated with polygenic scores for autism**

Varun Warrier and Simon Baron-Cohen

Autism Research Centre, Department of Psychiatry,

University of Cambridge

Correspondence to: Varun Warrier ([vw260@medschl.cam.ac.uk](mailto:vw260@medschl.cam.ac.uk)) or Simon Baron-Cohen ([sb205@cam.ac.uk](mailto:sb205@cam.ac.uk))

`

**What is this study about?**

We know that autism is partly genetic, and several genes and genetic variants contribute to the probability of being diagnosed with autism. Having higher number of genetic variants associated with autism does not necessarily mean a person has or needs a diagnosis of autism, but it may increase how many autistic traits that person has.

Previous studies have identified that autistic individuals report elevated levels of childhood trauma, self-harm, and suicidal ideation compared to the general population.

Here, using data from more than 100,000 individuals from the United Kingdom, we investigated if the number of genetic variants associated with autism a person has – termed their ‘genetic score’ for autism - is correlated with self-reported and retrospective measures of childhood trauma, self-harm behaviour and suicidal ideation.

**What did the study find?**

We identified that individuals who carry higher number of genetic variants associated with autism are more likely to report childhood trauma and self-harm behaviour and suicidal ideation.

It is important to remember that this study is correlational in design and so cannot be used to draw any causal conclusions. In particular, it would be inaccurate to conclude that a higher genetic score for autism causes childhood trauma, self-harm or suicidal ideation.

We surmise that the causal pathway may be more likely that those who carry a genetic predisposition for autism/higher autistic traits are not being adequately safe-guarded and are therefore more at risk for childhood trauma and self-harm.

It is also important to keep in mind that those individuals who carry the highest genetic score for autism, i.e. in the top centile, still have only a modest risk of reporting either childhood trauma and self-harm and suicidal ideation. For instance, individuals in the highest centile of genetic score for autism had a 28% increased risk of reporting childhood trauma compared to individuals in the lowest centile. Similarly, for self-harm and suicidal ideation, individuals in the highest centile had a 34% increased risk compared to individuals in the lowest centile. These numbers may seem big, but given that not many people report very high levels of either childhood trauma or self-harm behaviour and suicidal ideation, the final risk is not necessarily very high.

The study also found that there is a small but significant interaction between having high genetic score for autism and childhood trauma to predict self-harm and suicidal ideation. In other words, in individuals with both high childhood trauma and high genetic score for autism, the risk for self-harm and suicidal ideation is higher than in individuals with just a high genetic score for autism, or just higher reported childhood trauma. Again, note that this increase in risk is quite small.

Finally, various social factors like social relationship satisfaction, frequency of socialization, depressive symptoms, and educational attainment mediate the relationship between genetic variants for autism and self-harm and suicidal ideation. Identifying mediating factors is important as it can suggest potential avenues to mitigate risk of self-harm and suicidal ideation in autistic individuals.

**Are there not multiple types of childhood trauma?**

This is correct. Childhood trauma can be measured in multiple ways, and there are multiple types of childhood trauma. This study looked at five measures of childhood trauma – emotional abuse, physical abuse, sexual abuse, physical neglect, and emotional neglect, which were measured retrospectively. The study found that there is an association between genetic variants associated with autism and emotional abuse, physical abuse, and emotional neglect, and, to a smaller degree, with sexual abuse. There was no association with physical neglect.

**These are self-reported, retrospective measures of trauma. Will they replicate with other, prospective measures of trauma?**

This is unclear. Retrospective measures of trauma are different from prospective measures of trauma reported by a caregiver or a social worker, and are only modestly correlated. However, this is not to say that either one is more valid than the other, but they will have their own biases.

For instance, someone could later in their life evaluate an event as being traumatic as they have a different framework to interpret these events. On the other hand, they could forget or misremember traumatic events. It is also possible that prospectively measured trauma via, say social service reports, may measure only the most severe type of trauma.

So, the short answer is that we don’t know if these results will replicate with other, prospective measures of trauma primarily because they provide very different types of information.

**Is this study saying that childhood trauma is partly genetic?**

This study did find that that the differences in people’s experiences of childhood trauma can be attributed partly to the differences in their genetics. Readers should keep in mind that the majority of the differences in childhood trauma is non-genetic. Genes do not directly shape childhood traumatic experiences. Instead, they contribute to how our brains develop, and how brains process information from the world. As a result, they partly shape our personalities and our vulnerabilities.

For example, individuals who are genetically predisposed to be more socially naïve may be more likely have a traumatic experience because others may be more likely to take advantage of them. Similarly, individuals who are genetically predisposed to experience difficulties in social interactions or who may be timid may be more likely to be bullied by others. Further, genetic predisposition may give rise to differences in personality that may also contribute to whether we evaluate an experience as being traumatic or not.

In sum, the factors that actually cause traumatic event are almost entirely extrinsic, either other people acting to harm a person, or a situation that is harmful. By the same token, society more broadly and social groups such as families and educational or workplaces can provide supportive environments that may prevent traumatic events from occurring in the first place. This is what is meant by safe-guarding.

Since not all of those who have very high genetic scores for autism report higher levels of trauma, it follows that the role of the environment can increase risk of trauma and can also protect against the risk of trauma, depending on the kind of environment. Expressed differently, it is important we don’t fall into the trap of blaming the victim.

**Similarly, is the study saying that self-harm and suicidal ideation are partly genetic?**

Once again these are complex processes that are a result of an individual’s biological predisposition interacting with their environment. Again, most of the differences in suicidal ideation and self-harm in the population can be attributed to the environment. However, how people respond to various external life-events vary based on their genetic vulnerability.

This again draws attention to the need for safe-guarding of vulnerable individuals which in this study are people who – even without a formal diagnosis of autism – carry more of the genetic variants associated with autism/have a higher number of autistic traits.

**If genetics account for only a small part of childhood trauma, self-harm, and suicidal ideation, why study it?**

It is true that genetics accounts for only a small part of the differences in all three of the measures. However, understanding that genetics makes a person more vulnerable to the effects of adverse life experiences is an important discovery as it has implications for how we safe-guard individuals. In this study, the genetic score for autism is a proxy for autistic traits, and is useful as autistic traits were not directly measured in the current study. In the future, we would recommend that a questionnaire or screening tool such as the Autism Spectrum Quotient (AQ) be used to identify those who might have a high number of autistic traits, to ask them what additional support and safe-guarding they might need.

**Further reading**

Cassidy, S, Bradley, P, Robinson, J, Allison, C, McHugh, M, & Baron-Cohen, S, (2014) Suicidal ideation and suicide plans or attempts in adults with Asperger’s syndrome attending a specialist diagnostic clinic: a clinical cohort study. The Lancet Psychiatry, **1**, 142–147.

Griffiths, S, Allison, C, Kenny, R, Holt, R, Smith, P, Baron‐Cohen, S, (2019) The Vulnerability Experiences Quotient (VEQ): A study of vulnerability, mental health and life satisfaction in autistic adults. Autism Research.
