# Supplementary material for: Childhood trauma, life-time self-harm, and suicidal behaviour and ideation are associated with polygenic scores for autism
Source: Mol Psychiatry. 2019 Oct 29;26(5):1670–84. doi: 10.1038/s41380-019-0550-x (PMC8159746; doi:10.1038/s41380-019-0550-x)
Supplement: Supplementary file 2 — Supplementary Information (Figures, Tables, and Text) [file 41380_2019_550_MOESM2_ESM.docx]

**Supplementary Material**

**Childhood trauma, life-time self-harm and suicidal behaviour and ideation are associated with polygenic scores for autism**

Varun Warrier and Simon Baron-Cohen

Autism Research Centre, Department of Psychiatry, University of Cambridge

Correspondence to: Varun Warrier ([vw260@medschl.cam.ac.uk](mailto:vw260@medschl.cam.ac.uk)) or Simon Baron-Cohen (sb205@cam.ac.uk)

Contents

[Section 1: Mediation analyses 3](#_Toc21461404)

[Section 2: Brief literature support for mediators used in the analyses 5](#_Toc21461405)

[Section 3: Quality control of the Simons Simplex Collection Cohort 7](#_Toc21461406)

[Section 4: GWAS of the three primary phenotypes 8](#_Toc21461407)

[Supplementary Figure 1: Histograms of the three primary variables included in the study 9](#_Toc21461408)

[Supplementary Figure 2: Histograms of child trauma items 10](#_Toc21461409)

[Supplementary Figure 3: Histograms SSBI items 11](#_Toc21461410)

[Supplementary Figure 4: Histograms of mediating variables 12](#_Toc21461411)

[Supplementary Figure 5: Histograms of polygenic scores 13](#_Toc21461412)

[Supplementary Figure 6: Scores on individual childhood trauma items based on centiles of polygenic scores 14](#_Toc21461413)

[Supplementary Figure 7: Scores on individual SSBI items based on centiles of polygenic scores 15](#_Toc21461414)

[Supplementary Figure 8: Manhattan and QQplots for childhood trauma 16](#_Toc21461415)

[Supplementary Figure 9: Manhattan and QQplots for Self-harm ideation 17](#_Toc21461416)

[Supplementary Figure 10: Manhattan and QQplots for Self-harm score 18](#_Toc21461417)

[Supplementary Figure 11: Genetic correlations between autism and the three primary phenotypes after accounting for the genetic effects of other phenotypes 19](#_Toc21461418)

[Supplementary Table 1: Correlation between the mediators 20](#_Toc21461419)

[Supplementary Table 2: Number of SNPs at each P-value threshold for the Autism PGS 21](#_Toc21461420)

[Supplementary Table 3: Effect of PGS for Alzheimer’s across the three primary phenotypes 22](#_Toc21461421)

[Supplementary Table 4: Effects of PGS for autism on the three primary phenotypes and individual items after excluding autistic individuals 23](#_Toc21461422)

[Supplementary Table 5: Results of the polygenic transmission disequilibrium tests 24](#_Toc21461423)

[Supplementary Table 6: Results of the Genomic SEM analyses 25](#_Toc21461424)

[Supplementary Table 7: Effect of polygenic scores on mediators 26](#_Toc21461425)

[Supplementary Table 8: Effect of mediators on the two SSBI variables 27](#_Toc21461426)

[Supplementary Table 9: Mediation effects 28](#_Toc21461427)

[Supplementary Table 10: Interaction effects 30](#_Toc21461428)

# Section 1: Mediation analyses

We investigated 7 phenotypes as mediating variables. These are:

1. *Depressive symptoms* (39,479 < N < 39,551): We developed a depressive symptoms score based on items in the UK Biobank that mapped onto the DSM-5 criteria for Major Depressive Disorder^1^. All items were recoded to 0 and 1. Scores ranged from 0 to 8 with 8 being the highest score. The included items are:
   1. Prolonged feelings of sadness
   2. Prolonged loss of interest in normal activities
   3. Changes in sleep
   4. Feelings of tiredness during worst episode of depression
   5. Feelings of worthlessness during worst period of depression
   6. Difficulty concentrating during worst depression
   7. Thoughts of death during worst depression
   8. Weight change during worst episode of depression
2. *Anxiety symptoms* (28,177 < N < 28,231): We developed an anxiety symptoms score based on items in the UK Biobank that mapped onto the DSM-5^1^ criteria for Generalized Anxiety Disorder. All items were recoded to 0 and 1. Scores ranged from 0 to 7. The items included are:
   1. Difficulty stopping worrying during worst period of anxiety
   2. Multiple worries during worst period of anxiety
   3. More irritable than usual during worst period of anxiety
   4. Keyed up or on edge during worst period of anxiety
   5. Easily tired during worst period of anxiety
   6. Difficulty concentrating during worst period of anxiety
   7. Frequent trouble falling or staying asleep during worst period of anxiety
3. *Friendship and family relationship dissatisfaction* (56,704 < N < 56,842): This was measured using the question – ‘In general how satisfied are you with your friendships/family relationships?’. Participants could choose between extremely happy (coded 0) to extremely unhappy (coded 5). Data was collected across three instances, and we used the earliest instance for each participant aggregating data across all three instances.
4. *Job dissatisfaction* (30,533 < N < 30,575): This was measured using the question – ‘In general how satisfied are you with the work that you do?’. Participants could choose between extremely happy (coded 0) to extremely unhappy (coded 5). We excluded participants who responded with ‘I am not employed’ as the UK Biobank is an ageing cohort and it is difficult to distinguish retired participants from unemployed participants. Data was collected across three instances, and we used the earliest instance for each participant aggregating data across all three instances.
5. *Frequency of friendship/family visits* (117,616 < N < 117,772): This was measured using the question – ‘How often do you visit friends or family or have them visit you?’ Participants could choose from extremely happy (coded 1) to no friends/family outside household (coded 7). Data was collected across three instances, and we used the earliest instance for each participant aggregating data across all three instances.
6. *Confiding relationship* (115,402 < N < 115,553): This was measured using the question: ‘Since I was sixteen... I have been in a confiding relationship’. Participants could choose from never true (coded 0) to very often true (coded 4) .
7. *Educational attainment* (103279 < N < 103,417): This was measured using the question: “Which of the following qualifications do you have?”. We excluded individuals who reported “Other professional qualifications”, “None of the above” or “Prefer not to answer”. We then reverse coded the qualifications so higher scores mapped onto higher educational attainment.
8. *Cognitive aptitude (93811 < N < 93935):* This was measured using the total fluid intelligence scores using both in-person and online testing. For individuals who had completed both in-person and online testing, we prioritized online testing as this was done closer to the date when the mental health questionnaire was completed. Scores ranged from 0 – 13.

We note that, typically for mediation analyses, a temporal ordering is required with the independent variable (polygenic scores) occurring before the mediating variable, which in turn occurs before the dependent variable (SSBI). Whilst genotypes are largely immutable and occur at the time of conception, due to the cross-sectional nature of the current data available in the UK Biobank, we are unable to clearly demonstrate the causal nature of the mediator in the current study. However, collecting longitudinal data in large numbers is difficult, and variables of interest should be prioritized to prevent participant fatigue. In such instances, a cross-sectional mediation analysis is helpful to prioritize variants for further analysis, justifying the mediation analysis in the current dataset. Mediation analyses were conducted using the Mediation package in R using 1000 simulations.

# Section 2: Brief literature support for mediators used in the analyses

*1.1 Depression/depressive symptoms and Anxiety/anxiety symptoms*

A history of psychiatric illness is the primary risk factor for SSBI, with the effect size being larger than any of the other socio-demographic factors. There has been consistent support for this in nation-wide studies^2,3^. Specifically, in autism, one study has demonstrated that a history of psychiatric illness is, in line with the general population, the single biggest risk factor for suicide^4^. A handful of studies have provided greater resolution by investigating specific risk factors for suicide among psychiatric conditions. A meta-analysis of autopsy reports of more than 3,000 individuals who died by suicide identified that affective disorders including depressive disorders were the most common diagnostic category among individuals who had died by suicide (diagnosed in 43.2% of individuals who had died by suicide)^5^. These results have been supported by other meta-analyses and systematic reviews^6,7^. Longitudinal studies of suicidal ideation have suggested that depressive symptoms/depression were the best predictors of suicidal ideation^8,9^. One study has demonstrated that depressive symptoms mediate the risk between autistic traits and SSBI^10^. In clinical and cross-sectional studies there is some support for anxiety as a risk factor for SSBI^11–13^. This is significant even after controlling for a number of co-occuring conditions^14^.This has also been supported by longitudinal studies that have controlled for other co-occuring conditions^12,15^. Both anxiety and depression are elevated in autistic individuals^16,17^. Compared to the general populations, autistic individuals are 4-times more likely to experience depression^18^. Similarly, approximately 40% of children and adolescents with autism are likely to have a co-occuring anxiety disorder^19^.

*1.2 Social factors*

Loneliness and social isolation have been strongly linked to SSBI. Two prominent models link positive social support to reduced SSBI. The buffering hypothesis suggests positive social supports buffers the deleterious effect of stressors. In contrast, the main effects model suggests that being better integrated in a social network will increase healthy behaviours through conformity to social norms^20^. In a meta-analysis of more than 300,000 participants, Holt-Lunstad and colleagues^20^ identified that participants with stronger social relationships had a 50% increase in survival. Several studies have demonstrated that social isolation and reduced social relationship satisfaction are risk factors for SSBI^21–23^. Specifically, nation-wide registers in Scandinavian countries allow for interrogation of risk factors for SSBI, limiting potential confounding factors in other cohort designs such as healthy volunteer bias. In a nation-wide longitudinal study from Denmark, being single was the second biggest risk factor for suicide after psychiatric illness^2^. These results were replicated in a nation-wide study in Sweden: the unadjusted Hazards Ratio was 1.59 for unmarried women and 1.75 for unmarried men^3^. Autistic individuals, on average, tend to report increased loneliness and feeling socially excluded^24^, and report difficulties in forming and maintaining friendships^25,26^. Autistic traits are positively correlated with increased loneliness and perceived social support^27^, and the effect of autistic traits on depression are partly mediated by loneliness and satisfaction with social support^27^. In this study we investigate two qualitative metrics of social support – family relationship satisfaction and friendship satisfaction. We additionally also investigate two quantitative measures of social support – Number of confiding relationships and frequency of friendship/family visits.

*1.4 Job satisfaction*

A few studies have investigated the role of job satisfaction/employment in SSBI. In a nation-wide register study from Denmark, unemployment modestly increased the risk for suicide (1.14 < OR < 1.24), with the risk increasing as the percentage of time being unemployed increased^2^. A smaller study in Sweden reported higher OR for unemployment as a risk factor for suicide (1.93 < OR < 3.86)^28^. In a larger, nation-wide study in Sweden, adjusted Hazard Ratios for suicide were 1.97 for unemployed men and 1.66 for unemployed women^3^. Unemployment is a significant issue in the autism community. According to a survey conducted by the National Autistic Society in the UK, only 16% of autistic adults are in full time employment, and less than a third are employed in some paid work (See: <https://www.autism.org.uk/get-involved/media-centre/news/2016-10-27-employment-gap.aspx>). Supporting this, a cross-sectional study in Germany identified that late-diagnosed autistic adults were disadvantaged at the job market^29^.

# Section 3: Quality control of the Simons Simplex Collection Cohort

We downloaded genotype data from the SSC from SFARI base (<https://www.sfari.org/resource/sfari-base/).> All participants provided informed consent or assent and ethical approval to conduct analyses on de-identified data was obtained from the University of Cambridge Human Biology Research Ethics Committee. Individuals were genotyped using three different arrays:  Illumina Omni2.5, Illumina 1Mv3, or Illumina 1Mv1. Informed consent or assent was obtained from all participants. We restricted individuals to those with a genotyping rate > 95%, were not heterozygosity outliers (within 3 SD from the mean), whose reported sex matched their genetic sex, and families with < 5% mendelian errors. %. We removed SNPs that significantly deviated from Hardy-Weinberg Equilibrium ( P < 1x10^-6^), had mendelian errors in more than 10% of the families, and SNPs that were not genotyped in more than 10% of the families. Families of primarily European ancestry were identified using multidimensional scaling using genetic data from parents, who are unrelated. Genetic principal components were calculated using only SNPs with minor allele frequency > 5%, and pruning the SNPs in Plink using an r^2^ of 0.2. We excluded families from further downstream analyses if either one the parents were greater or less than 5 standard deviations from the means of the first two genetic principal components calculated using only the unrelated individuals in HapMap3 CEU and TSI populations. Quality control was done using Plink v 1.9 and R. Phasing and imputation was conducted using the Michigan Imputation Server (https://imputationserver.sph.umich.edu/start.html) using the 1000 genomes Phase 3 v5 as the reference panel. The full pipeline is available here: <https://github.com/autism-research-centre/SSC_liftover_imputation>.

# Section 4: GWAS of the three primary phenotypes

We conducted genome-wide association analysis using BOLT-LMM^87^, which uses a mixed linear effects model to control for relatedness whilst increasing statistical power. We restricted our analyses to SNPs with a minor allele frequency > 0.1%, which are in Hardy-Weinberg Equilibrium (P > 1 x 10^-6^), and have a genotyping rate > 0.9. We included only SNPs with an imputation R^2^ > 0.4. Subsequently, analyses were conducted for 16,754,618 SNPs. We excluded individuals who were not of self-reported European Ancestry, or were 5 SDs away from the mean of the first and second genetic principal components in the self-reported European ancestry subset, had a genotyping rate < 90%, were of discordant sex (reported sex did not match genetic sex), and who were outliers for heterozygosity. This resulted in 144,278 individuals for the two SSBI GWAS (63,025 males, 81,253 females) and 143,473 individuals for the childhood trauma GWAS (62,868 males and 80,608 females). In the GWAS analyses, we included age, sex, the first 20 genetic principal components, and genotype batch as covariates. To calculate relatedness among individuals, we included 1 million SNPs with minor allele frequency > 1%, had a Hardy-Weinberg Equilibrium P-value > 1x10^-6^, an imputation R^2^ > 0.9, and genotyping rate > 0.9 in BOLT-LMM^88^.

# Supplementary Figure 1: Histograms of the three primary variables included in the study


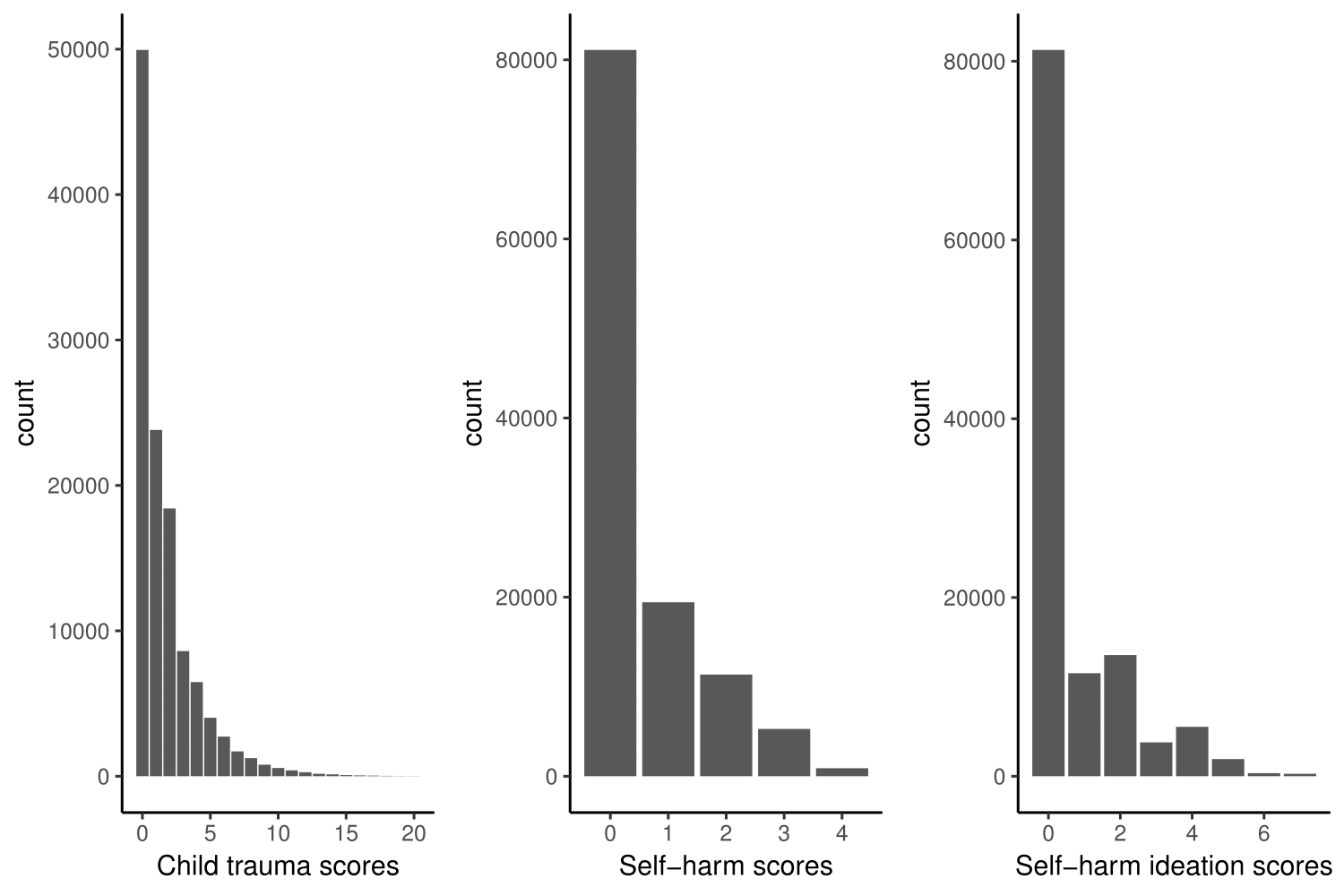


*Frequency histogram of the three primary variables included in the study.*

# Supplementary Figure 2: Histograms of child trauma items


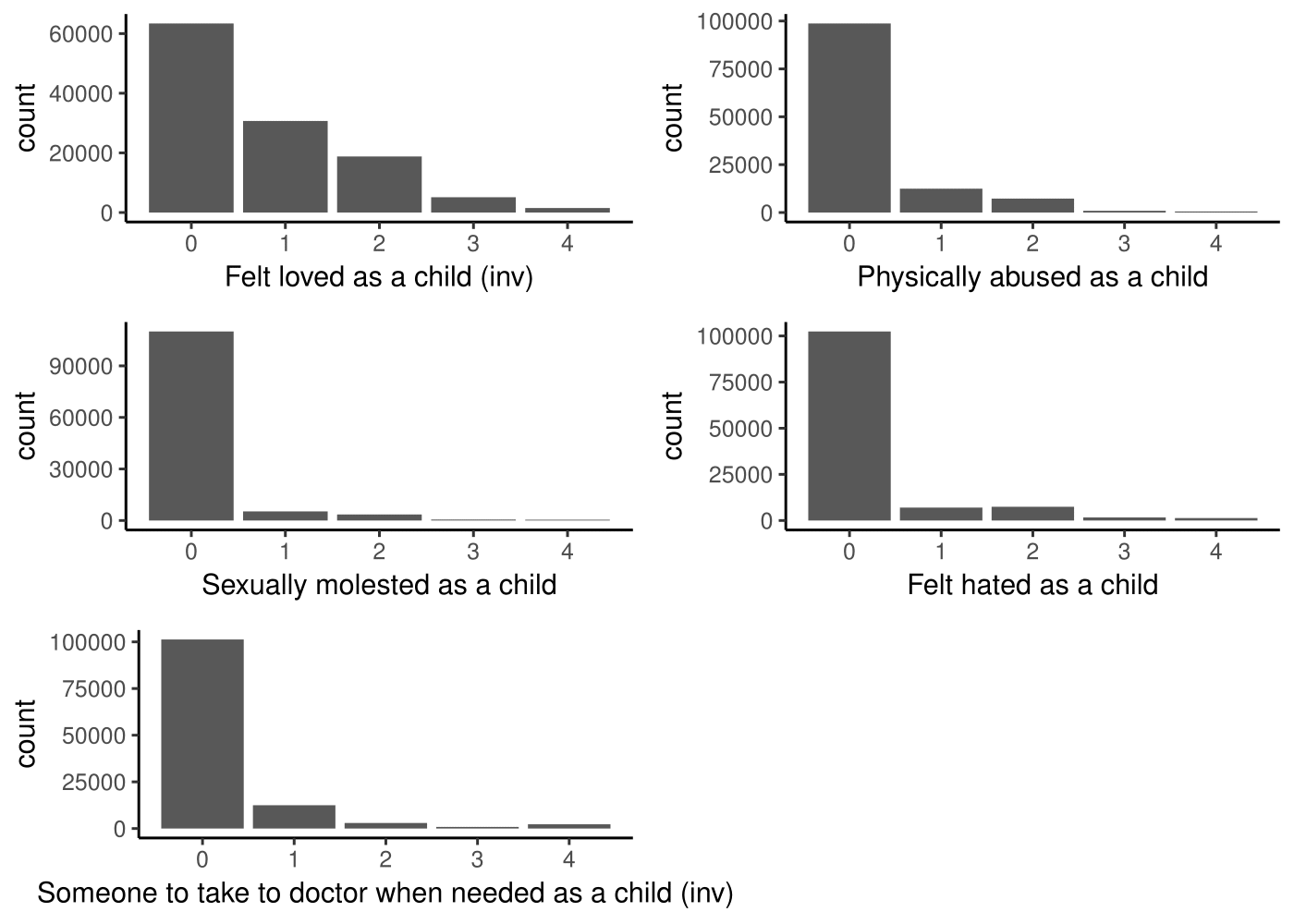


*Frequency histogram of the five individual items included in the child trauma phenotype.*

# Supplementary Figure 3: Histograms SSBI items


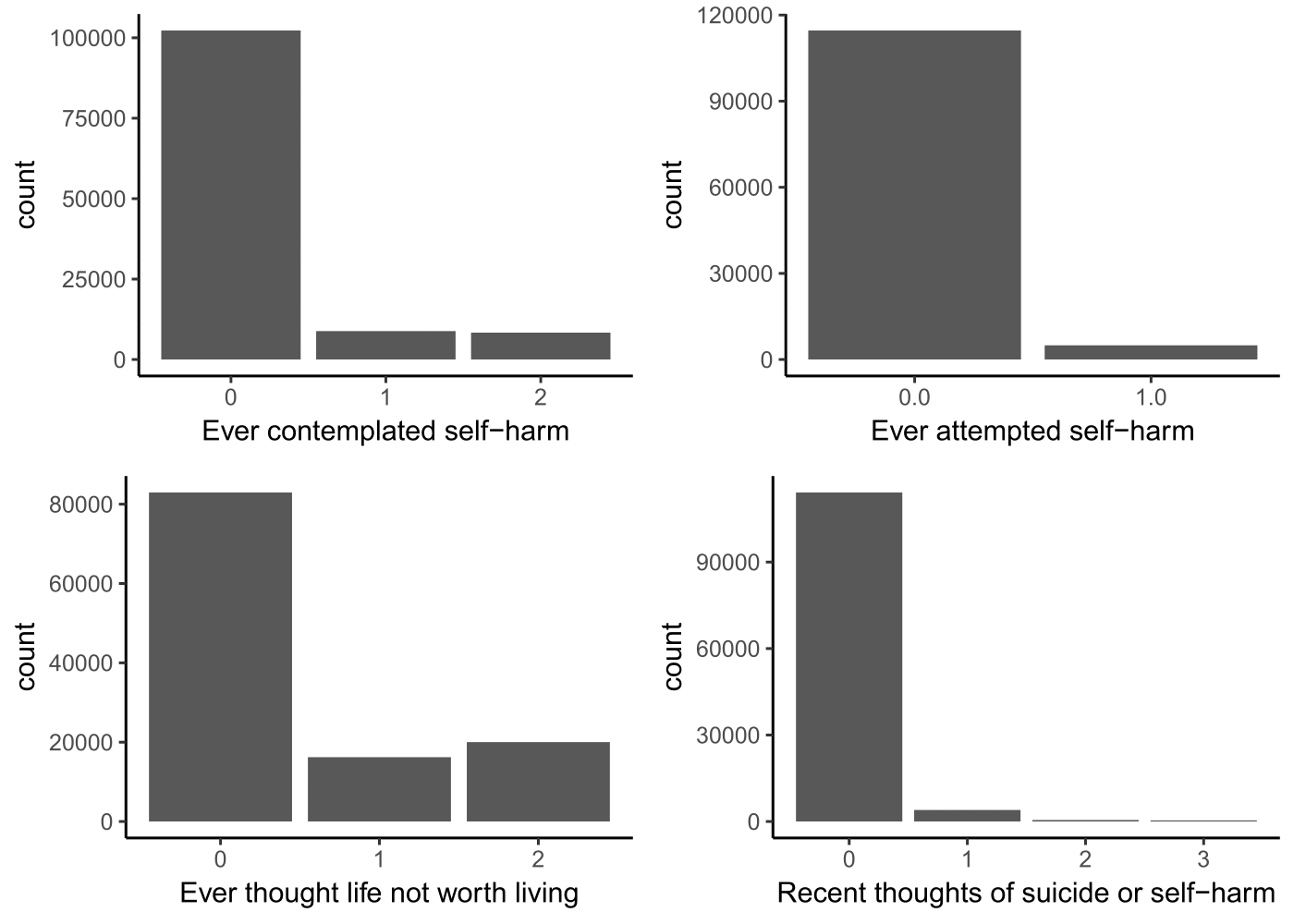
 *Frequency histogram of the four individual items included in the SSBI phenotypes.*

# Supplementary Figure 4: Histograms of mediating variables


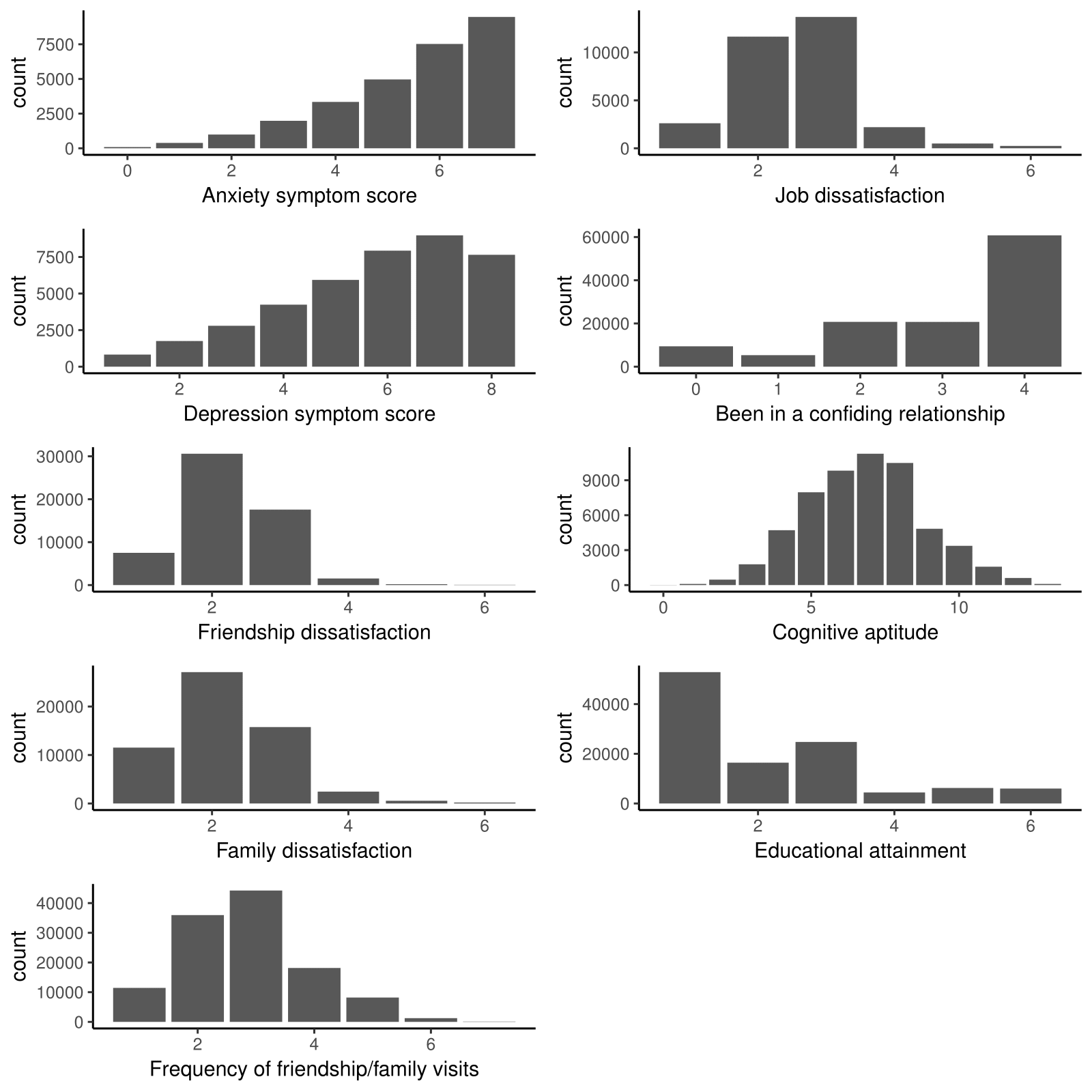


*Frequency histogram of the nine mediating variables.*

# Supplementary Figure 5: Histograms of polygenic scores


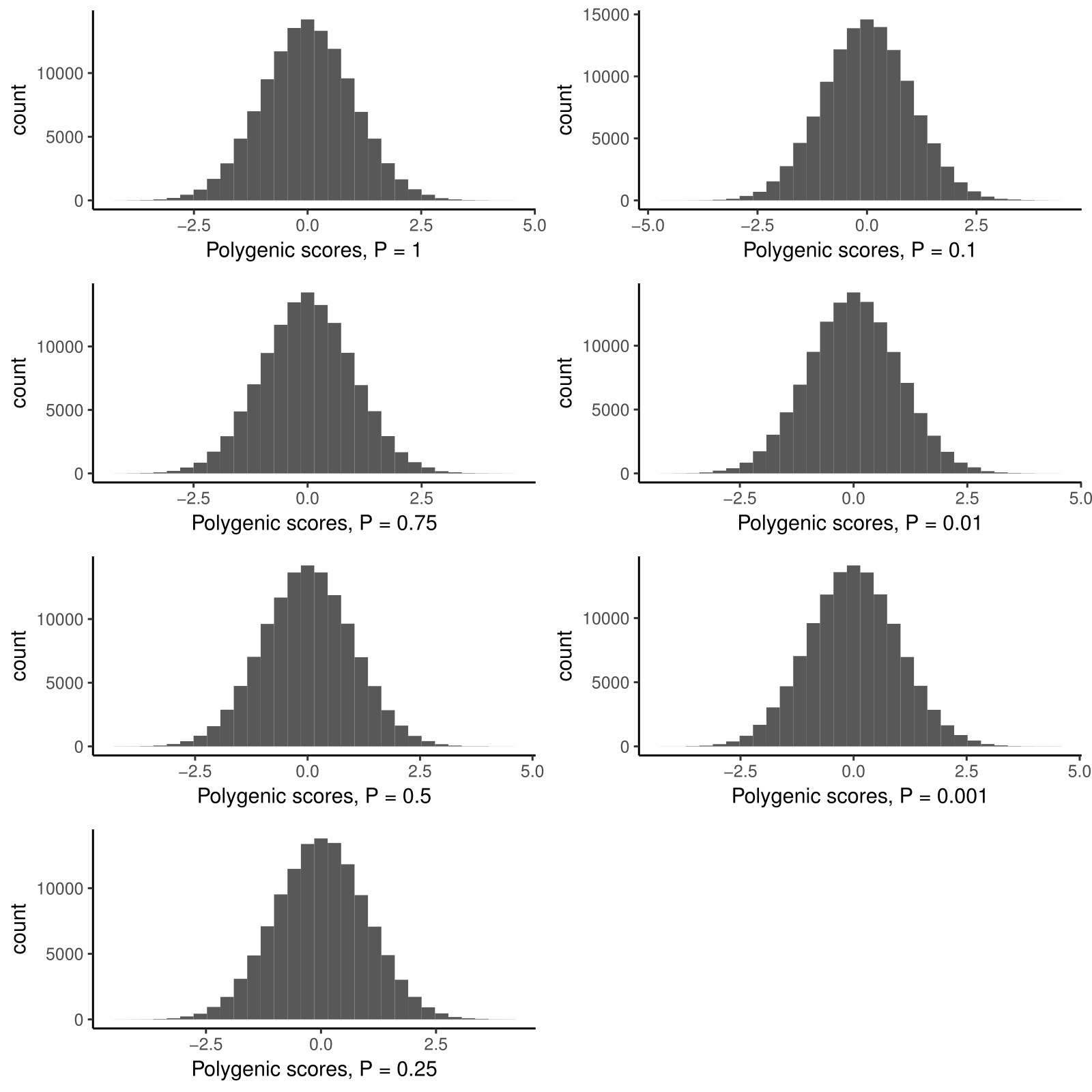


*Frequency histogram of standardized autism polygenic scores in the UK Biobank.*

# Supplementary Figure 6: Scores on individual childhood trauma items based on centiles of polygenic scores


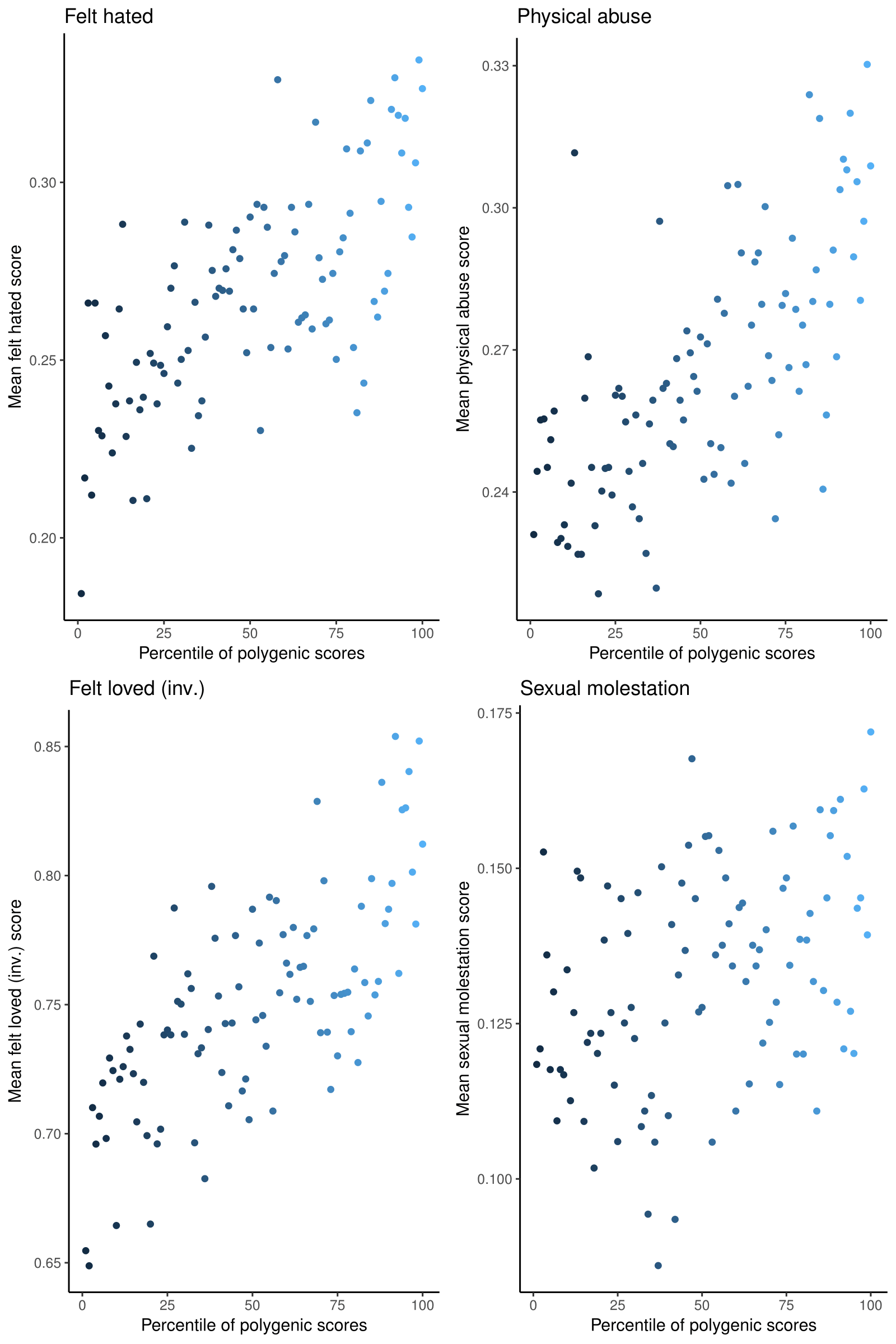


*This figure provides the scores of four individual trauma items against the percentile of polygenic scores after the cohort was divided into 100 groups based on polygenic scores. Each dot in the plot represents an average phenotypic score for that group. Please note, ‘Felt loved’ is inverse scored.*

# Supplementary Figure 7: Scores on individual SSBI items based on centiles of polygenic scores


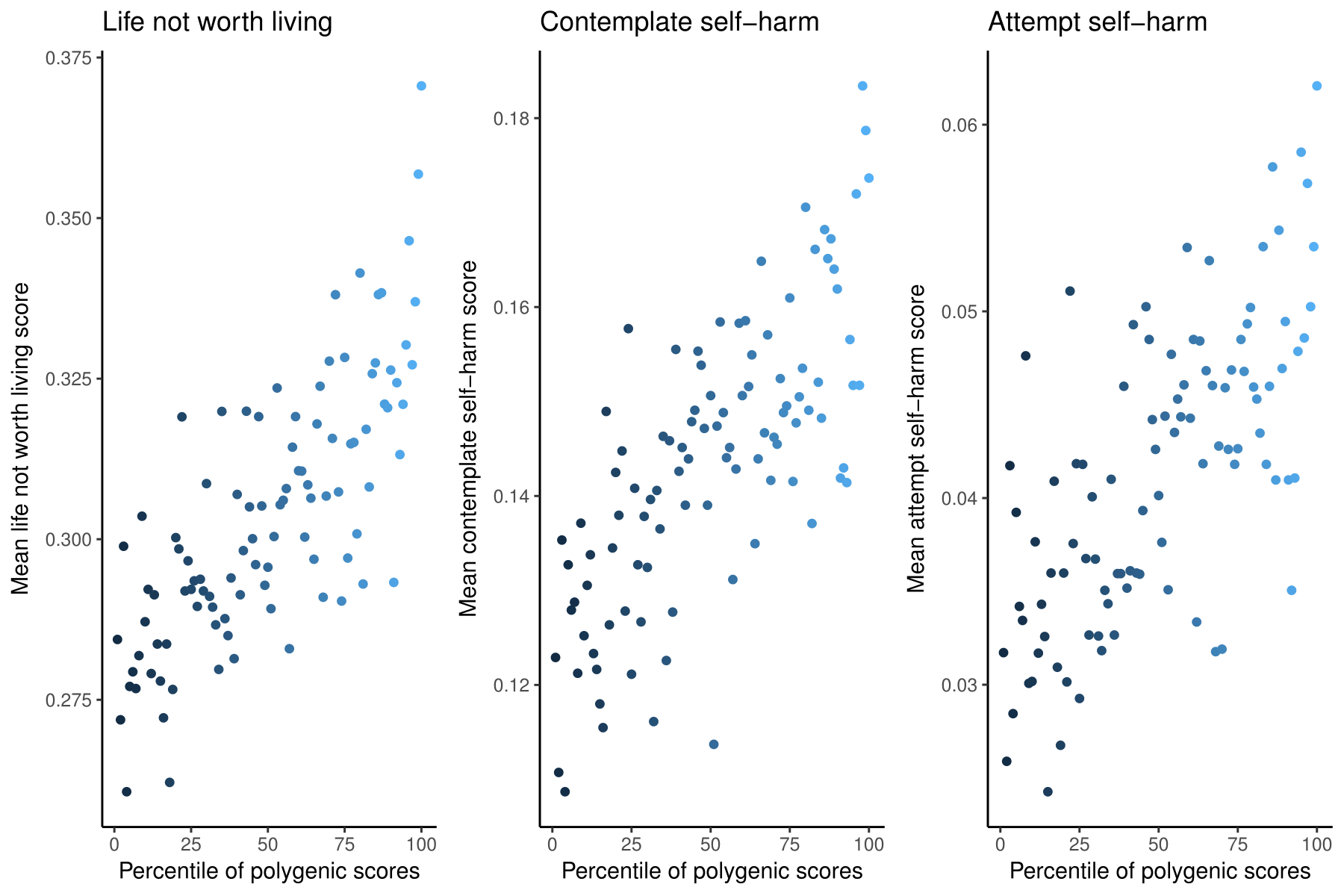


*This figure provides the scores of three individual SSBI item against the percentile of polygenic scores after the cohort was divided into 100 groups based on polygenic scores. Each dot in the plot represents an average phenotypic score for that group.*

# Supplementary Figure 8: Manhattan and QQplots for childhood trauma


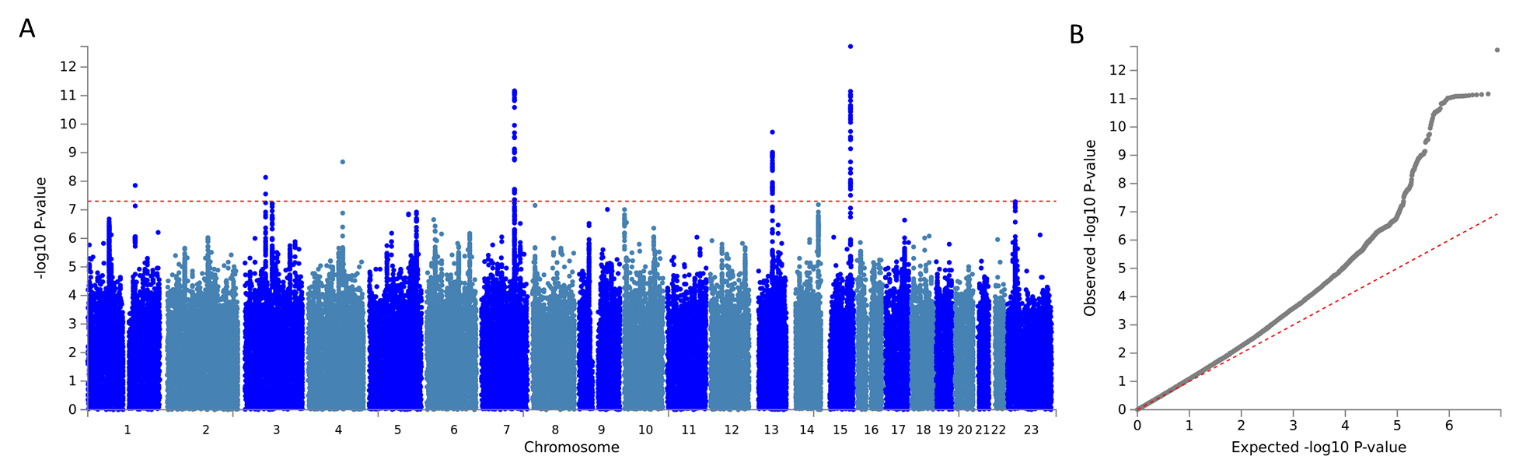


8A: Manhattan plot for childhood trauma. 8B. QQ plot for childhood trauma. Childhood trauma had a SNP heritability of 0.0834±0.0049, P < 2.2x10^-16^. We did not identify a significant inflation in test statistics due to unaccounted population stratification as measured by LD-score intercept: 1.0088±0.0071.

# Supplementary Figure 9: Manhattan and QQplots for Self-harm ideation


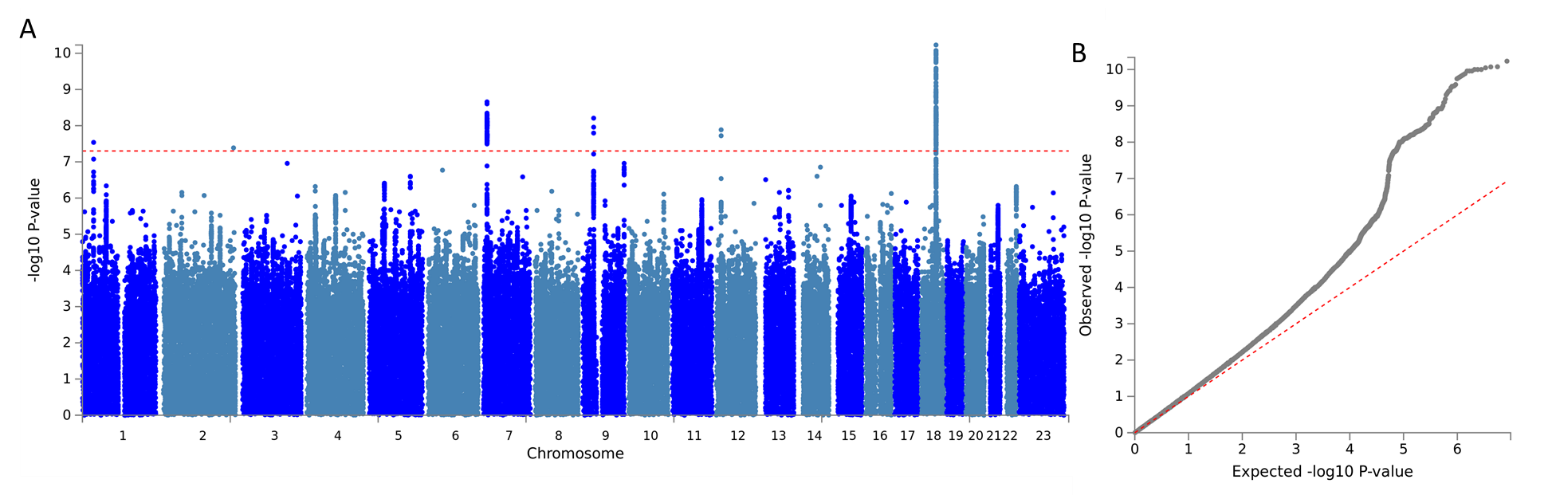


9A: Manhattan plot for childhood trauma. 9B. QQ plot for self-harm ideation. Self-harm ideation had a SNP heritability of 0.071±0.0047, P < 2.2x10^-16^. We did not identify a substantial inflation in test statistics due to unaccounted population stratification as measured by LD-score intercept: 1.014±0.0067.

# Supplementary Figure 10: Manhattan and QQplots for Self-harm score


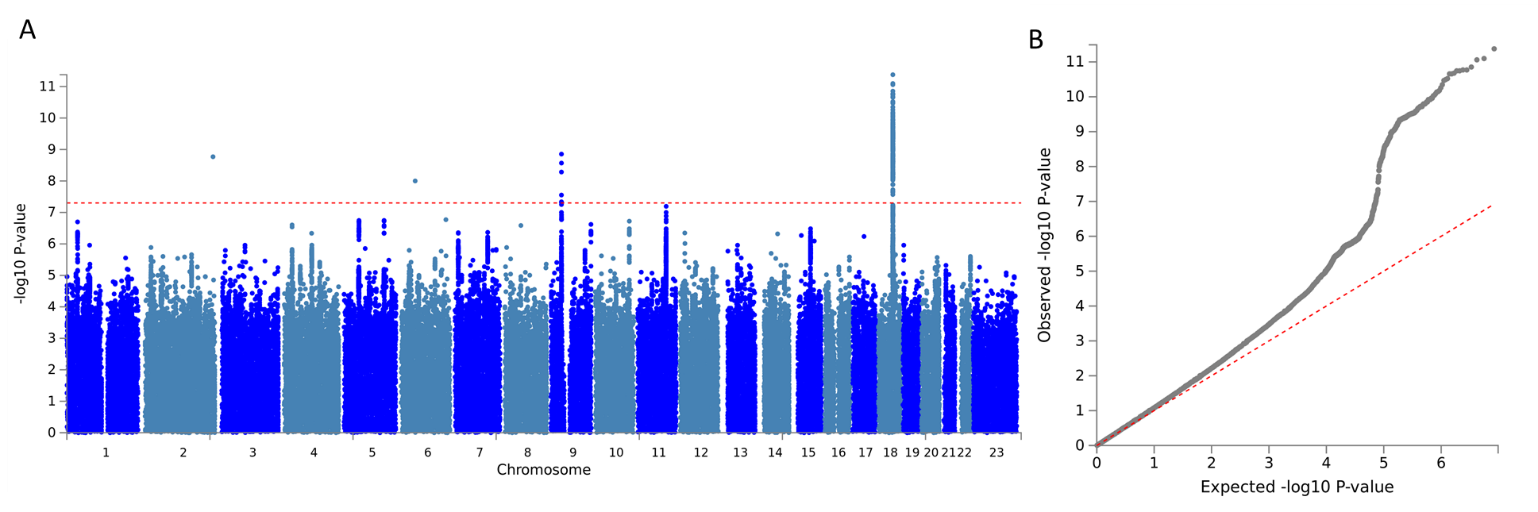


10A: Manhattan plot for childhood trauma. 10B. QQ plot for self-harm score. Self-harm score had a SNP heritability of 0.071±0.0047, P < 2.2x10^-16^. We did not identify a substantial inflation in test statistics due to unaccounted population stratification as measured by LD-score intercept: 1.010±0.0067.

# Supplementary Figure 11: Genetic correlations between autism and the three primary phenotypes after accounting for the genetic effects of other phenotypes


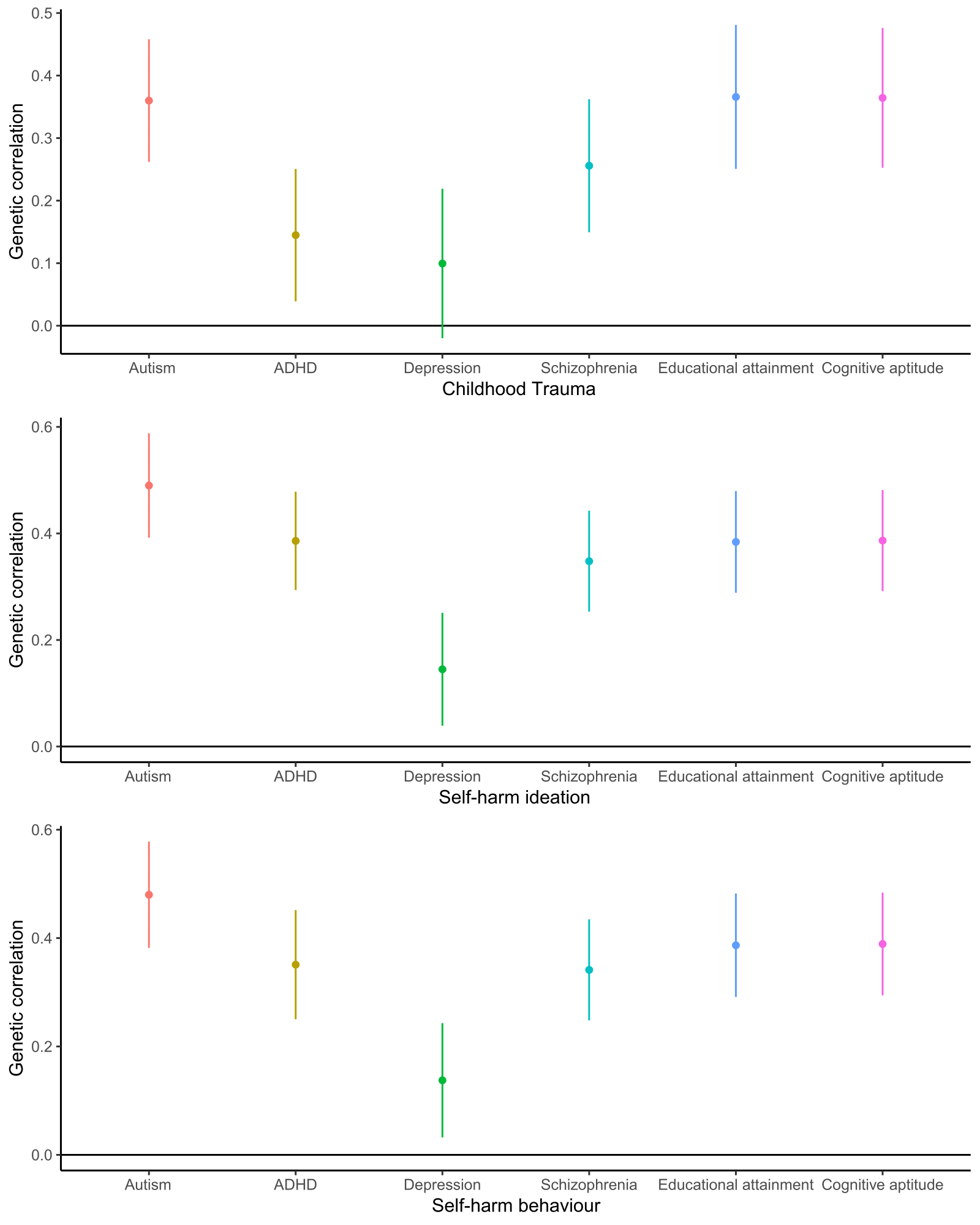


The graphs above provide point estimates of genetic correlation and 95% confidence intervals for the three primary phenotypes. We first provide the genetic correlation with Autism. All subsequent genetic correlations are the between the primary phenotype and autism after conditioning on the effects of second phenotype which is indicated on the x axis (ADHD, Depression, Schizophrenia, Educational attainment and Cognitive aptitude).

# Supplementary Table 1: Correlation between the mediators

|  | Depression score | Family relationship dissatisfaction | Friendship relationship dissatisfaction | Frequency of friendship/family visit | Educational attainment |
| --- | --- | --- | --- | --- | --- |
| Depression score | 1 | 0.083 | 0.055 | -0.017 | 0.038 |
| Family relationship  dissatisfaction | 0.1 | 1 | 0.467 | 0.078 | -0.023 |
| Friendship relationship  dissatisfaction | 0.067 | 0.49 | 1 | 0.134 | -0.048 |
| Frequency of friendship/family visit | -0.018 | 0.1 | 0.162 | 1 | -0.082 |
| Educational attainment | 0.033 | -0.024 | -0.049 | -0.082 | 1 |

*Correlation between the five variables included in the mediation analyses. The top triangle (in red) provides Kendall’s Tau Rank correlation coefficients. The bottom triangle provides Pearson’s correlation coefficients. All correlations are statistically significant (P < 0.001).*

# Supplementary Table 2: Number of SNPs at each P-value threshold for the Autism PGS

| Threshold | N SNPs |
| --- | --- |
| 0.001 | 1406 |
| 0.01 | 8734 |
| 0.1 | 57382 |
| 0.25 | 119197 |
| 0.5 | 200889 |
| 0.75 | 261065 |
| 1 | 300133 |

*This table provides the number of SNPs (LD-clumped, R^2^ = 0.2) for each P-value threshold for the autism polygenic scores.*

# Supplementary Table 3: Effect of PGS for Alzheimer’s across the three primary phenotypes

| Beta | SE | Z | P | P-threshold | R^2^ (%) | Phenotype |
| --- | --- | --- | --- | --- | --- | --- |
| 4.40E-03 | 2.90E-03 | 1.51 | 0.13 | 1 | 0.001 | Childhood trauma |
| 4.42E-03 | 2.90E-03 | 1.52 | 0.13 | 0.75 | 0.0011 | Childhood trauma |
| 4.49E-03 | 2.90E-03 | 1.55 | 0.12 | 0.5 | 0.0011 | Childhood trauma |
| 3.64E-03 | 2.90E-03 | 1.25 | 0.21 | 0.25 | 0.0004 | Childhood trauma |
| 1.53E-03 | 2.90E-03 | 0.52 | 0.60 | 0.1 | -0.0006 | Childhood trauma |
| 1.60E-03 | 2.89E-03 | 0.55 | 0.58 | 0.01 | -0.0006 | Childhood trauma |
| -3.38E-03 | 2.89E-03 | -1.17 | 0.24 | 0.001 | 0.0003 | Childhood trauma |
| -3.85E-03 | 2.88E-03 | -1.33 | 0.18 | 1 | 0.001 | Self-harm ideation |
| -3.98E-03 | 2.88E-03 | -1.38 | 0.17 | 0.75 | 0.001 | Self-harm ideation |
| -3.69E-03 | 2.88E-03 | -1.28 | 0.20 | 0.5 | 0.001 | Self-harm ideation |
| -5.07E-03 | 2.88E-03 | -1.76 | 0.08 | 0.25 | 0.002 | Self-harm ideation |
| -5.63E-03 | 2.88E-03 | -1.95 | 0.05 | 0.1 | 0.002 | Self-harm ideation |
| -1.19E-03 | 2.87E-03 | -0.41 | 0.68 | 0.01 | -0.001 | Self-harm ideation |
| -2.46E-03 | 2.87E-03 | -0.85 | 0.39 | 0.001 | 0 | Self-harm ideation |
| -3.08E-03 | 2.88E-03 | -1.07 | 0.28 | 1 | 0 | Self-harm score |
| -3.25E-03 | 2.88E-03 | -1.12 | 0.26 | 0.75 | 0 | Self-harm score |
| -2.96E-03 | 2.88E-03 | -1.03 | 0.30 | 0.5 | 0 | Self-harm score |
| -4.19E-03 | 2.88E-03 | -1.45 | 0.15 | 0.25 | 0.001 | Self-harm score |
| -4.17E-03 | 2.88E-03 | -1.45 | 0.15 | 0.1 | 0.001 | Self-harm score |
| -1.61E-03 | 2.87E-03 | -0.56 | 0.57 | 0.01 | -0.001 | Self-harm score |
| -2.02E-03 | 2.87E-03 | -0.71 | 0.48 | 0.001 | -0.001 | Self-harm score |

*This table provides the result of the polygenic score analyses for the three primary phenotypes at various 7 different P-value thresholds. For each analysis we report the regression coefficient (Beta) and the accompanying standard errors (SE), Z-score (Z) and P-value of the Z-score (P). Variance explained (R^2^) is provided in percentages.*

# Supplementary Table 4: Effects of PGS for autism on the three primary phenotypes and individual items after excluding autistic individuals

| Item | Beta | SE | Z | P | R^2^ (%) | P-threshold | Category |
| --- | --- | --- | --- | --- | --- | --- | --- |
| Childhood trauma | 3.00E-02 | 2.86E-03 | 10.708 | < 2E-16 | 0.094 | 1 | Primary phenotype |
| Self-harm score | 3.61E-02 | 2.86E-03 | 12.612 | < 2E-16 | 0.12 | 0.75 | Primary phenotype |
| Self-harm ideation | 3.29E-02 | 2.86E-03 | 11.479 | < 2E-16 | 0.1 | 0.75 | Primary phenotype |
| felt loved as child | 3.06E-02 | 2.89E-03 | 10.578 | < 2E-16 | 0.09 | 1 | Childhood trauma |
| sexually molested | 1.11E-02 | 2.87E-03 | 3.851 | 1.18E-04 | 0.063 | 1 | Childhood trauma |
| physically abused | 2.51E-02 | 2.87E-03 | 8.729 | < 2E-16 | 0.011 | 1 | Childhood trauma |
| felt hated as a child | 2.65E-02 | 2.87E-03 | 9.23 | < 2E-16 | 0.069 | 1 | Childhood trauma |
| taken to the doctor | 1.69E-03 | 2.88E-03 | 0.585 | 5.58E-01 | -0.0006 | 1 | Childhood trauma |
| thought life not worth living | 3.26E-02 | 2.86E-03 | 11.383 | < 2E-16 | 0.16 | 0.75 | SSBI |
| contemplated self-harm | 3.10E-02 | 2.86E-03 | 10.836 | < 2E-16 | 0.14 | 0.75 | SSBI |
| ever attempted self-harm | 2.61E-02 | 2.87E-03 | 9.078 | < 2E-16 | 0.1 | 0.75 | SSBI |
| recent thoughts of suicide and self-harm | 7.48E-03 | 2.89E-03 | 2.59 | 9.61E-03 | 0.008 | 0.75 | SSBI |

*This table provides the result of the polygenic score analyses for the three primary phenotypes and individuals items in the UK Biobank after excluding autistic individuals. We chose the P-value threshold that explained the maximum variance in the initial analyses which did not exclude autistic individuals. For each analysis we report the regression coefficient (Beta) and the accompanying standard errors (SE), Z-score (Z) and P-value of the Z-score (P). Variance explained (R^2^) is provided in percentages.*

# Supplementary Table 5: Results of the polygenic transmission disequilibrium tests

| Category | Case mean | Case SD | P | Sibling mean | Sibling SD | P |
| --- | --- | --- | --- | --- | --- | --- |
| Self-harm score | 6.4E-02 | 2.16E-02 | 2.6E-03 | -3.4E-02 | 2.3E-02 | 0.14 |
| childhood trauma | 4.8E-02 | 2.11E-02 | 2.2E-02 | 1.7E-02 | 2.30E-02 | 0.45 |
| Self-harm ideation | 6.5E-02 | 2.13E-02 | 2.2E-03 | -3.0E-02 | 2.39E-02 | 0.20 |

*This table provides the results of the pTDT. Case mean represents the mean deviation of polygenic scores from the mid-parent polygenic scores (standardized on the mid-parent polygenic scores). Sibling mean represents the mean deviation of the polygenic scores from the mid-parent polygenic scores (standardized on the mid-parent polygenic scores). We provide the standard deviation for the deviation and the P-values.*

# Supplementary Table 6: Results of the Genomic SEM analyses

| r_g_ | SE | Z | P | Covariate | Phenotype |
| --- | --- | --- | --- | --- | --- |
| 0.36 | 0.05 | 7.20 | 8.13E-11 | None | Childhood trauma |
| 0.14 | 0.05 | 2.68 | 3.64E-03 | ADHD | Childhood trauma |
| 0.10 | 0.06 | 1.63 | 1.02E-01 | Depression | Childhood trauma |
| 0.26 | 0.05 | 4.71 | 2.45E-06 | Schizophrenia | Childhood trauma |
| 0.37 | 0.06 | 6.23 | 4.59E-10 | Educational attainment | Childhood trauma |
| 0.36 | 0.06 | 6.39 | 1.65E-10 | Cognitive aptitude | Childhood trauma |
|  |  |  |  |  |  |
| 0.49 | 0.05 | 9.80 | 4.17E-21 | None | self-harm ideation |
| 0.39 | 0.05 | 8.20 | 2.45E-16 | ADHD | self-harm ideation |
| 0.15 | 0.05 | 2.68 | 7.26E-03 | Depression | self-harm ideation |
| 0.35 | 0.05 | 7.20 | 6.19E-13 | Schizophrenia | self-harm ideation |
| 0.38 | 0.05 | 7.88 | 3.22E-15 | Educational attainment | self-harm ideation |
| 0.39 | 0.05 | 7.98 | 1.44E-15 | Cognitive aptitude | self-harm ideation |
|  |  |  |  |  |  |
| 0.48 | 0.05 | 9.60 | 4.58E-21 | None | self-harm score |
| 0.35 | 0.05 | 6.83 | 8.50E-12 | ADHD | self-harm score |
| 0.14 | 0.05 | 2.56 | 1.05E-02 | Depression | self-harm score |
| 0.34 | 0.05 | 7.18 | 6.75E-13 | Schizophrenia | self-harm score |
| 0.39 | 0.05 | 7.94 | 2.04E-15 | Educational attainment | self-harm score |
| 0.39 | 0.05 | 8.05 | 8.29E-16 | Cognitive aptitude | self-harm score |

*Genetic correlations between autism and the three primary phenotypes without accounting for other phenotypes (None) and after accounting for ADHD, Depression, Schizophrenia, Educational Attainment, and Cognitive Aptitude using genomic structural equation modelling.* *r_g_ is the genetic correlation, SE is the Standard Error, Z is the Z-value and P is the P-value.*

# Supplementary Table 7: Effect of polygenic scores on mediators

| Phenotype | Beta | SE | Z | P |
| --- | --- | --- | --- | --- |
| Anxiety symptoms | 1.24E-02 | 5.84E-03 | 2.12 | 0.033 |
| Depression symptoms | 1.73E-02 | 4.81E-03 | 3.60 | 3.14E-04 |
| Friendship dissatisfaction | 1.38E-02 | 4.16E-03 | 3.32 | 8.92E-04 |
| Family relationship dissatisfaction | 1.91E-02 | 4.17E-03 | 4.57 | 4.68E-06 |
| Frequency of friendship/family visit | 1.45E-02 | 2.84E-03 | 5.10 | 3.31E-07 |
| Job dissatisfaction | 1.13E-02 | 5.66E-03 | 2.00 | 0.045 |
| Confiding relationship | -7.17E-03 | 0.002924 | -2.45 | 0.01 |
| Educational attainment | 1.51E-02 | 3.21E-03 | 4.70 | 2.54E-06 |
| Cognitive aptitude | 1.43E-02 | 3.08E-03 | 4.65 | 3.22E-06 |

*This table provides the regression estimates of the autism PGS on the 9 mediators. Beta is the regression estimate, SE is the Standard Error, Z is the Z-value and P is the P-value. For the regression, we included age, sex, the first twenty genetic principal components, and array as covariates, all of which (except for array) were standardized.*

# Supplementary Table 8: Effect of mediators on the two SSBI variables

| Mediator | Estimate | SE | Z | P | Category |
| --- | --- | --- | --- | --- | --- |
| Confiding relationship | -1.08E-01 | 2.89E-03 | -37.29 | < 2E-16 | self-harm score |
| Frequency of friendship/family visit | 4.79E-02 | 2.92E-03 | 16.37 | < 2E-16 | self-harm score |
| Job dissatisfaction | 1.19E-01 | 5.83E-03 | 20.49 | < 2E-16 | self-harm score |
| Family relationship dissatisfaction | 1.56E-01 | 4.11E-03 | 38.02 | < 2E-16 | self-harm score |
| Friendship dissatisfaction | 1.29E-01 | 4.13E-03 | 31.19 | < 2E-16 | self-harm score |
| Depressive symptoms | 4.98E-01 | 5.57E-03 | 89.35 | < 2E-16 | self-harm score |
| Anxiety symptoms | 3.10E-01 | 7.17E-03 | 43.2 | < 2E-16 | self-harm score |
| Educational attainment | 2.16E-02 | 3.11E-03 | 6.951 | 3.6E-12 | self-harm score |
| Cognitive aptitude | 2.69E-03 | 2.25E-03 | 0.828 | 0.4075 | self-harm score |
| Confiding relationship | -1.11E-01 | 2.89E-03 | -38.25 | < 2E-16 | self-harm ideation |
| Frequency of friendship/family visit | 5.05E-02 | 2.92E-03 | 17.28 | < 2E-16 | self-harm ideation |
| Job dissatisfaction | 1.36E-01 | 5.83E-03 | 23.37 | < 2E-16 | self-harm ideation |
| Family relationship dissatisfaction | 1.63E-01 | 4.11E-03 | 39.66 | < 2E-16 | self-harm ideation |
| Friendship dissatisfaction | 1.38E-01 | 4.13E-03 | 33.40 | < 2E-16 | self-harm ideation |
| Depressive symptoms | 5.12E-01 | 5.70E-03 | 89.87 | < 2E-16 | self-harm ideation |
| Anxiety symptoms | 3.34E-01 | 7.42E-03 | 44.99 | < 2E-16 | self-harm ideation |
| Educational attainment | 2.74E-02 | 3.11E-03 | 8.81 | < 2E-16 | self-harm ideation |
| Cognitive aptitude | 1.00E-02 | 3.25E-03 | 3.07 | 0.0021 | self-harm ideation |

*This table provides the regression estimates of the 9 mediators on the two SSBI phenotypes. Beta is the regression estimate, SE is the Standard Error, Z is the Z-value and P is the P-value. For the regression, we included age, sex, the first twenty genetic principal components, and array as covariates, all of which (except for the array) were standardized.*

# Supplementary Table 9: Mediation effects

| Depression symptoms | | | | | |
| --- | --- | --- | --- | --- | --- |
|  | Estimate | 95% CI Lower | 95% CI Upper | P | Phenotype |
| ACME | 0.00893 | 0.00388 | 0.01 | < 2E-16 | Self-harm ideation |
| ADE | 0.02955 | 0.0180 | 0.04 | < 2E-16 | Self-harm ideation |
| Total Effect | 0.03848 | 0.02617 | 0.05 | < 2E-16 | Self-harm ideation |
| Prop. Mediated | 0.23165 | 0.11182 | 0.37 | < 2E-16 | Self-harm ideation |
|  | Estimate | 95% CI Lower | 95% CI Upper | P |  |
| ACME | 0.00888 | 0.00418 | 0.01 | < 2E-16 | Self-harm |
| ADE | 0.03363 | 0.02343 | 0.04 | < 2E-16 | Self-harm |
| Total Effect | 0.04251 | 0.03110 | 0.05 | < 2E-16 | Self-harm |
| Prop. Mediated | 0.20942 | 0.09973 | 0.32 | < 2E-16 | Self-harm |
| Family dissatisfaction | | | | | |
|  | Estimate | 95% CI Lower | 95% CI Upper | P | Phenotype |
| ACME | 0.00298 | 0.00178 | 0.004 | < 2E-16 | Self-harm ideation |
| ADE | 0.02923 | 0.02081 | 0.04 | < 2E-16 | Self-harm ideation |
| Total Effect | 0.03222 | 0.02307 | 0.04 | < 2E-16 | Self-harm ideation |
| Prop. Mediated | 0.09179 | 0.05652 | 0.14 | < 2E-16 | Self-harm ideation |
|  | Estimate | 95% CI Lower | 95% CI Upper | P |  |
| ACME | 0.00287 | 0.00157 | 0.004 | < 2E-16 | Self-harm |
| ADE | 0.03244 | 0.02471 | 0.04 | < 2E-16 | Self-harm |
| Total Effect | 0.03531 | 0.02720 | 0.04 | < 2E-16 | Self-harm |
| Prop. Mediated | 0.08133 | 0.04473 | 0.13 | < 2E-16 | Self-harm |
| Friendship dissatisfaction | | | | | |
|  | Estimate | 95% CI Lower | 95% CI Upper | P | Phenotype |
| ACME | 0.001766 | 0.000687 | 0.002 | < 2E-16 | Self-harm ideation |
| ADE | 0.030325 | 0.022430 | 0.04 | < 2E-16 | Self-harm ideation |
| Total Effect | 0.032090 | 0.024486 | 0.04 | < 2E-16 | Self-harm ideation |
| Prop. Mediated | 0.055374 | 0.021366 | 0.09 | < 2E-16 | Self-harm ideation |
|  | Estimate | 95% CI Lower | 95% CI Upper | P |  |
| ACME | 0.001656 | 0.000609 | 0.003 | < 2E-16 | Self-harm |
| ADE | 0.033702 | 0.025942 | 0.04 | < 2E-16 | Self-harm |
| Total Effect | 0.035359 | 0.027482 | 0.04 | < 2E-16 | Self-harm |
| Prop. Mediated | 0.046759 | 0.016686 | 0.08 | < 2E-16 | Self-harm |
| Frequency of friendship/family visits | | | | | |
|  | Estimate | 95% CI Lower | 95% CI Upper | P | Phenotype |
| ACME | 0.000730 | 0.000437 | 0.0009 | < 2E-16 | Self-harm ideation |
| ADE | 0.032428 | 0.026674 | 0.04 | < 2E-16 | Self-harm ideation |
| Total Effect | 0.033158 | 0.027387 | 0.04 | < 2E-16 | Self-harm ideation |
| Prop. Mediated | 0.022064 | 0.012726 | 0.03 | < 2E-16 | Self-harm ideation |
|  | Estimate | 95% CI Lower | 95% CI Upper | P |  |
| ACME | 0.000688 | 0.000419 | 0.009 | < 2E-16 | Self-harm |
| ADE | 0.035655 | 0.030356 | 0.04 | < 2E-16 | Self-harm |
| Total Effect | 0.036342 | 0.031171 | 0.04 | < 2E-16 | Self-harm |
| Prop. Mediated | 0.018985 | 0.011295 | 0.03 | < 2E-16 | Self-harm |
| Education | | | | | |
|  | Estimate | 95% CI Lower | 95% CI Upper | P | Phenotype |
| ACME | 0.000390 | 0.000225 | 0.00055 | < 2E-16 | Self-harm ideation |
| ADE | 0.032358 | 0.02667 | 0.04 | < 2E-16 | Self-harm ideation |
| Total Effect | 0.032748 | 0.026995 | 0.04 | < 2E-16 | Self-harm ideation |
| Prop. Mediated | 0.011733 | 0.006504 | 0.02 | < 2E-16 | Self-harm ideation |
|  | Estimate | 95% CI Lower | 95% CI Upper | P |  |
| ACME | 0.000305 | 0.000157 | 0.000453 | < 2E-16 | Self-harm |
| ADE | 0.035361 | 0.029305 | 0.04 | < 2E-16 | Self-harm |
| Total Effect | 0.035666 | 0.02577 | 0.04 | < 2E-16 | Self-harm |
| Prop. Mediated | 0.008447 | 0.00436 | 0.01 | < 2E-16 | Self-harm |

*ACME: Average Causal Mediated Effect, ADE: Average Direct Effect (i.e. proportion not mediated). Please note, the analysis does not provide causality, and we use the term ACME as it is standard in mediation analyses.*

# Supplementary Table 10: Interaction effects

| Beta | SE | Z | P | P-threshold | Variable | Category |
| --- | --- | --- | --- | --- | --- | --- |
| 4.14E-02 | 3.88E-03 | 10.68 | <2.2E-16 | 1 | PGS | Childhood Trauma Score |
| -7.13E-02 | 5.82E-03 | -12.24 | <2.2E-16 | 1 | Sex (Male) | Childhood Trauma Score |
| -2.31E-02 | 5.80E-03 | -3.98 | 6.74E-05 | 1 | PGS:Sex (Male) | Childhood Trauma Score |
| 3.88E-02 | 3.86E-03 | 10.06 | <2.2E-16 | 0.75 | PGS | Self-harm ideation score |
| -1.22E-01 | 5.78E-03 | -21.09 | <2.2E-16 | 0.75 | Sex (Male) | Self-harm ideation score |
| -1.27E-02 | 5.76E-03 | -2.21 | 0.026 | 0.75 | PGS:Sex (Male) | Self-harm ideation score |
| 4.26E-02 | 3.85E-03 | 11.07 | <2.2E-16 | 0.75 | PGS | Self-harm score |
| -1.49E-01 | 5.78E-03 | -25.81 | <2.2E-16 | 0.75 | Sex (Male) | Self-harm score |
| -1.42E-02 | 5.76E-03 | -2.47 | 0.013 | 0.75 | PGS:Sex (Male) | Self-harm score |
| 2.40E-02 | 2.75E-03 | 8.77 | <2.2E-16 | 0.75 | PGS | Self-harm ideation score |
| 2.86E-01 | 2.77E-03 | 103.34 | <2.2E-16 | 0.75 | Childtrauma | Self-harm ideation score |
| 7.47E-03 | 2.75E-03 | 2.71 | 0.006 | 0.75 | PGS:Childtrauma | Self-harm ideation score |
| 2.73E-02 | 2.75E-03 | 9.93 | <2.2E-16 | 0.75 | PGS | Self-harm score |
| 2.84E-01 | 2.78E-03 | 102.25 | <2.2E-16 | 0.75 | Childtrauma | Self-harm score |
| 8.37E-03 | 2.76E-03 | 3.03 | 0.002 | 0.75 | PGS:Childtrauma | Self-harm score |

*This table provides the results of the interaction effect for the three primary phenotypes. For all three phenotypes, we tested the interaction between PGS and sex. Additionally, for the two SSBI phenotypes, we tested the interaction between PGS and childhood trauma. For each interaction, we report the two main effects and the interaction effect.* *For each analysis we report the regression coefficient (Beta) and the accompanying standard errors (SE), Z-score (Z) and P-value of the Z-score (P).*

**References**

1. American Psychiatric Association. *The Diagnostic and Statistical Manual (5th Ed.)*. Washington, DC; 2013.

2. Qin P, Agerbo E, Mortensen PB. Suicide Risk in Relation to Socioeconomic, Demographic, Psychiatric, and Familial Factors: A National Register–Based Study of All Suicides in Denmark, 1981–1997. *Am J Psychiatry*. 2003;160(4):765-772. doi:10.1176/appi.ajp.160.4.765

3. Crump C, Sundquist K, Sundquist J, Winkleby MA. Sociodemographic, psychiatric and somatic risk factors for suicide: a Swedish national cohort study. *Psychol Med*. 2014;44(02):279-289. doi:10.1017/S0033291713000810

4. Hirvikoski T, Mittendorfer-Rutz E, Boman M, Larsson H, Lichtenstein P, Bölte S. Premature mortality in autism spectrum disorder. *Br J Psychiatry*. 2016;208(3):232-238. doi:10.1192/bjp.bp.114.160192

5. Arsenault-Lapierre G, Kim C, Turecki G. Psychiatric diagnoses in 3275 suicides: a meta-analysis. *BMC Psychiatry*. 2004;4(1):37. doi:10.1186/1471-244X-4-37

6. Cavanagh JTO, Carson AJ, Sharpe M, Lawrie SM. Psychological autopsy studies of suicide: a systematic review. *Psychol Med*. 2003;33(3):395-405. http://www.ncbi.nlm.nih.gov/pubmed/12701661. Accessed October 30, 2018.

7. Yoshimasu K, Kiyohara C, Miyashita K, Stress Research Group of the Japanese Society for Hygiene TSRG of the JS for. Suicidal risk factors and completed suicide: meta-analyses based on psychological autopsy studies. *Environ Health Prev Med*. 2008;13(5):243-256. doi:10.1007/s12199-008-0037-x

8. GARRISON CZ, ADDY CL, JACKSON KL, McKEOWN RE, WALLER JL. A Longitudinal Study of Suicidal Ideation in Young Adolescents. *J Am Acad Child Adolesc Psychiatry*. 1991;30(4):597-603. doi:10.1097/00004583-199107000-00011

9. Prinstein MJ, Nock MK, Simon V, Aikins JW, Cheah CSL, Spirito A. Longitudinal trajectories and predictors of adolescent suicidal ideation and attempts following inpatient hospitalization. *J Consult Clin Psychol*. 2008;76(1):92-103. doi:10.1037/0022-006X.76.1.92

10. Culpin I, Mars B, Pearson RM, et al. Autistic Traits and Suicidal Thoughts, Plans, and Self-Harm in Late Adolescence: Population-Based Cohort Study. *J Am Acad Child Adolesc Psychiatry*. 2018;57(5):313-320.e6. doi:10.1016/J.JAAC.2018.01.023

11. Karakoç Demirkaya S, Tutkunkardaş MD, Mukaddes NM. Assessment of suicidality in children and adolescents with diagnosis of high functioning autism spectrum disorder in a Turkish clinical sample. *Neuropsychiatr Dis Treat*. 2016;Volume 12:2921-2926. doi:10.2147/NDT.S118304

12. Sareen J, Cox BJ, Afifi TO, et al. Anxiety Disorders and Risk for Suicidal Ideation and Suicide Attempts. *Arch Gen Psychiatry*. 2005;62(11):1249. doi:10.1001/archpsyc.62.11.1249

13. Cox BJ, Direnfeld DM, Swinson RP, Norton GR. Suicidal ideation and suicide attempts in panic disorder and social phobia. *Am J Psychiatry*. 1994;151(6):882-887. doi:10.1176/ajp.151.6.882

14. Nepon J, Belik S-L, Bolton J, Sareen J. The relationship between anxiety disorders and suicide attempts: findings from the National Epidemiologic Survey on Alcohol and Related Conditions. *Depress Anxiety*. 2010;27(9):791-798. doi:10.1002/da.20674

15. BODEN JM, FERGUSSON DM, JOHN HORWOOD L. Anxiety disorders and suicidal behaviours in adolescence and young adulthood: findings from a longitudinal study. *Psychol Med*. 2007;37(03):431. doi:10.1017/S0033291706009147

16. Strang JF, Kenworthy L, Daniolos P, et al. Depression and Anxiety Symptoms in Children and Adolescents with Autism Spectrum Disorders without Intellectual Disability. *Res Autism Spectr Disord*. 2012;6(1):406-412. doi:10.1016/j.rasd.2011.06.015

17. Kim JA, Szatmari P, Bryson SE, Streiner DL, Wilson FJ. The Prevalence of Anxiety and Mood Problems among Children with Autism and Asperger Syndrome. *Autism*. 2000;4(2):117-132. doi:10.1177/1362361300004002002

18. Hudson CC, Hall L, Harkness KL. Prevalence of Depressive Disorders in Individuals with Autism Spectrum Disorder: a Meta-Analysis. *J Abnorm Child Psychol*. March 2018:1-11. doi:10.1007/s10802-018-0402-1

19. van Steensel FJA, Bögels SM, Perrin S. Anxiety disorders in children and adolescents with autistic spectrum disorders: a meta-analysis. *Clin Child Fam Psychol Rev*. 2011;14(3):302-317. doi:10.1007/s10567-011-0097-0

20. Holt-Lunstad J, Smith TB, Layton JB. Social Relationships and Mortality Risk: A Meta-analytic Review. Brayne C, ed. *PLoS Med*. 2010;7(7):e1000316. doi:10.1371/journal.pmed.1000316

21. Santini ZI, Koyanagi A, Tyrovolas S, Haro JM. The association of relationship quality and social networks with depression, anxiety, and suicidal ideation among older married adults: Findings from a cross-sectional analysis of the Irish Longitudinal Study on Ageing (TILDA). *J Affect Disord*. 2015;179:134-141. doi:10.1016/j.jad.2015.03.015

22. Chang Q, Chan CH, Yip PSF. A meta-analytic review on social relationships and suicidal ideation among older adults. *Soc Sci Med*. 2017;191:65-76. doi:10.1016/j.socscimed.2017.09.003

23. Lasgaard M, Goossens L, Elklit A. Loneliness, Depressive Symptomatology, and Suicide Ideation in Adolescence: Cross-Sectional and Longitudinal Analyses. *J Abnorm Child Psychol*. 2011;39(1):137-150. doi:10.1007/s10802-010-9442-x

24. Deckers A, Muris P, Roelofs J. Being on Your Own or Feeling Lonely? Loneliness and Other Social Variables in Youths with Autism Spectrum Disorders. *Child Psychiatry Hum Dev*. 2017;48(5):828-839. doi:10.1007/s10578-016-0707-7

25. Kasari C, Locke J, Gulsrud A, Rotheram-Fuller E. Social Networks and Friendships at School: Comparing Children With and Without ASD. *J Autism Dev Disord*. 2011;41(5):533-544. doi:10.1007/s10803-010-1076-x

26. Rotheram-Fuller E, Kasari C, Chamberlain B, Locke J. Social involvement of children with autism spectrum disorders in elementary school classrooms. *J Child Psychol Psychiatry*. 2010;51(11):1227-1234. doi:10.1111/j.1469-7610.2010.02289.x

27. Hedley D, Uljarević M, Foley K-R, Richdale A, Trollor J. Risk and protective factors underlying depression and suicidal ideation in Autism Spectrum Disorder. *Depress Anxiety*. 2018;35(7):648-657. doi:10.1002/da.22759

28. Johansson S-E, Sundquist J. Unemployment is an important risk factor for suicide in contemporary Sweden: an 11-year follow-up study of a cross-sectional sample of 37 789 people. *Public Health*. 1997;111(1):41-45. doi:10.1038/SJ.PH.1900317

29. Frank F, Jablotschkin M, Arthen T, et al. Education and employment status of adults with autism spectrum disorders in Germany - a cross-sectional-survey. *BMC Psychiatry*. 2018;18(1):75. doi:10.1186/s12888-018-1645-7

30. Bycroft C, Freeman C, Petkova D, et al. The UK Biobank resource with deep phenotyping and genomic data. *Nature*. 2018;562(7726):203-209. doi:10.1038/s41586-018-0579-z

31. Purcell S, Neale B, Todd-Brown K, et al. PLINK: a tool set for whole-genome association and population-based linkage analyses. *Am J Hum Genet*. 2007;81(3):559-575. doi:10.1086/519795

32. Euesden J, Lewis CM, O’Reilly PF. PRSice: Polygenic Risk Score software. *Bioinformatics*. 2015;31(9):1466-1468. doi:10.1093/bioinformatics/btu848

33. Grove J, Ripke S, Als TD, et al. Common risk variants identified in autism spectrum disorder. *bioRxiv*. November 2017:224774. doi:10.1101/224774

34. Lambert J-C, Ibrahim-Verbaas CA, Harold D, et al. Meta-analysis of 74,046 individuals identifies 11 new susceptibility loci for Alzheimer’s disease. *Nat Genet*. 2013;45(12):1452-1458. doi:10.1038/ng.2802

35. St Pourcain B, Robinson EB, Anttila V, et al. ASD and schizophrenia show distinct developmental profiles in common genetic overlap with population-based social communication difficulties. *Mol Psychiatry*. January 2017. doi:10.1038/mp.2016.198
